# Stat3/Cdc25a-dependent cell proliferation promotes embryonic axis extension during zebrafish gastrulation

**DOI:** 10.1371/journal.pgen.1006564

**Published:** 2017-02-21

**Authors:** Yinzi Liu, Diane S. Sepich, Lilianna Solnica-Krezel

**Affiliations:** Department of Developmental Biology, Washington University School of Medicine, St. Louis, Missouri, United States of America; University of Pennsylvania School of Medicine, UNITED STATES

## Abstract

Cell proliferation has generally been considered dispensable for anteroposterior extension of embryonic axis during vertebrate gastrulation. Signal transducer and activator of transcription 3 (Stat3), a conserved controller of cell proliferation, survival and regeneration, is associated with human scoliosis, cancer and Hyper IgE Syndrome. Zebrafish Stat3 was proposed to govern convergence and extension gastrulation movements in part by promoting Wnt/Planar Cell Polarity (PCP) signaling, a conserved regulator of mediolaterally polarized cell behaviors. Here, using zebrafish *stat3* null mutants and pharmacological tools, we demonstrate that cell proliferation contributes to anteroposterior embryonic axis extension. Zebrafish embryos lacking maternal and zygotic Stat3 expression exhibit normal convergence movements and planar cell polarity signaling, but transient axis elongation defect due to insufficient number of cells resulting largely from reduced cell proliferation and increased apoptosis. Pharmacologic inhibition of cell proliferation during gastrulation phenocopied axis elongation defects. Stat3 regulates cell proliferation and axis extension in part via upregulation of Cdc25a expression during oogenesis. Accordingly, restoring Cdc25a expression in *stat3* mutants partially suppressed cell proliferation and gastrulation defects. During later development, *stat3* mutant zebrafish exhibit stunted growth, scoliosis, excessive inflammation, and fail to thrive, affording a genetic tool to study Stat3 function in vertebrate development, regeneration, and disease.

## Author summary

During vertebrate embryogenesis, cell proliferation, fate specification and cell movements are key processes that transform a fertilized egg into an embryo with head, trunk and tail. Cell proliferation is orchestrated by maternal and zygotic functions of conserved regulators including Cdc25a, and has generally been considered dispensable for embryonic axis elongation. Stat3 transcriptional factor, a known promoter of cell proliferation, is associated with human scoliosis, inflammation and cancer. Based on morpholino-mediated downregulation of Stat3 during zebrafish embryogenesis, Stat3 was previously proposed to regulate convergence and extension cell movements that narrow the embryonic body and elongate it from head to tail partially through planar cell polarity signaling and unknown transcriptional targets. Here, we report that zebrafish mutants lacking maternal and zygotic Stat3 expression exhibit normal convergence movements and planar cell polarity signaling, but transient axis elongation defect due to insufficient number of cells resulting largely from reduced cell proliferation and increased cell death. Accordingly, pharmacologic inhibition of cell proliferation also hinders axis elongation. Further experiments indicate that Stat3 promotes head- to -tail axis elongation by stimulating cell proliferation in part via upregulation of Cdc25a expression during oogenesis. During later development, zebrafish *stat3* mutants exhibit scoliosis and inflammation, potentially affording a new tool to study related human diseases.

## Introduction

Signal transducer and activator of transcription 3 (STAT3) is an essential mediator of cytokine and growth factor signaling involved in animal development, homeostasis and disease [1, 2]. Primarily a transcription factor, STAT3 activates or inhibits expression of downstream genes involved in cell proliferation, apoptosis, stem cell maintenance, differentiation, and migration in normal tissues. Non-transcriptional functions of STAT3 in microtubule, mitochondria, and chromatin regulation have also been reported [3, 4]. In tumors, constitutively active STAT3 drives cell proliferation through upregulation of cell cycle-regulators such as c-Myc and Cyclin D, promotes pluripotency of cancer stem cells, and potentiates metastasis by modulating cytoskeleton and extracellular matrix [4, 5]. Further underscoring its role in human disease, dominant autosomal *STAT3* mutations account for numerous symptoms in Hyper-IgE syndrome (HIES) patients such as misregulated TNFα levels and scoliosis [6–8]. Disruption of murine *Stat3* in hematopoietic cells causes Crohn’s disease-like immunodeficiency [9].

Stat3 also has essential functions in development. Firstly, STAT signaling regulates border cell migration in the developing *Drosophila* egg chamber [10]. Secondly, *Stat3* knockout mice die by early gastrulation [11], implying critical but yet undefined roles of Stat3 in embryogenesis. Thirdly, morpholino studies in zebrafish indicated a requirement for Stat3 in planar cell polarity (PCP) signaling and gastrulation movements [12, 13]. Later during development, Stat3 promotes bone formation, as its deletion in mouse osteoclasts and osteoblasts decreased bone density and volume [14, 15]. Dominant negative and morpholino-interference approaches also implicated zebrafish Stat3 in heart and eye regeneration [16, 17].

Here, we report analyses of zebrafish *stat3* mutants that lead us to propose a different model wherein Stat3 regulates embryonic axis extension by ensuring sufficient number of cells through its role as promoter of cell proliferation during blastula and gastrula stages and cell survival during gastrulation. The cell cycle during zebrafish embryogenesis is complex. Early embryos undergo rapid and synchronous cell cleavages [18] consisting of DNA synthesis (S) and mitosis (M) phases without transcription or cell growth. After mid-blastula transition (MBT) and activation of the zygotic genome, cell cycles slow down and become asynchronous with the acquisition of a G2 phase but little growth [19]. Conserved from fly to mammals, Cdc25a phosphatase is a positive regulator of cell cycle progression during embryogenesis [20–23]. Through activation of Cyclin B/Cdk1 complexes, Cdc25a synthesized from both maternal and zygotic transcripts propels mitotic entry [23]. But how Cdc25a is activated in early vertebrate embryos is unclear.

MBT is followed by gastrulation, during which cells engage in large-scale migrations and rearrangements to establish future body plan. Convergence and extension (C&E) are conserved gastrulation movements that narrow the germ layers mediolaterally and lengthen them along the anteroposterior (AP) axis [24]. Under the influence of Wnt/PCP signaling, cells become mediolaterally elongated and either migrate dorsally (convergence) or engage in polarized intercalations that preferentially separate anterior and posterior neighbors to drive simultaneous mediolateral (ML) convergence and AP axis extension [24, 25]. Disruption of Wnt/PCP signaling in zebrafish mutants such as *silberblick* (*slb*)/*wnt11* and *trilobite* (*tri*)/*vangl2* impairs ML cell elongation and polarized cell behaviors, consequently producing a shorter and wider embryonic body [26, 27]. Interestingly, impaired cell elongation and ML alignment, and consequently defective C&E were also reported in *stat3* morphants, implicating Stat3 as a regulator of PCP signaling during zebrafish C&E [12, 13].

Cell proliferation and gastrulation movements must be coordinated to achieve proper embryogenesis. Indeed, rapid cell proliferation usually precedes gastrulation, during which cell divisions occur infrequently [28]. Gastrulating cells divide at the expense of migration by rounding up and abolishing their planar polarized asymmetries [29], likely because cell division and motility utilize common cytoskeletal machineries. Limiting cell divisions is required for normal C&E of the paraxial mesoderm in *Xenopus* [28] and posterior body elongation in zebrafish [30]. Conversely, cell proliferation appears dispensable for axis elongation during gastrulation. Complete gastrulation featuring elongated bodies occurs in the zebrafish *emi* mutants in which mitosis ceases from early gastrulation, and in embryos where cell proliferation is chemically inhibited during gastrulation [31, 32]. However, without quantitative assessment of C&E movements in these embryos, some contribution of cell proliferation to gastrulation cannot be excluded. In addition, the relationship between cell division orientation and axis elongation in zebrafish remains unresolved. While some studies pointed out that oriented cell division under the regulation of Wnt/PCP signaling is a driving force for axis elongation [33], others argued against the importance of cell division orientation in axis extension [32].

Here, we report that Stat3 contributes to extension movements during zebrafish gastrulation by ensuring sufficient number of cells through its role as a promoter of cell proliferation and survival. Investigating null *stat3* mutations generated with transcription activator-like effector nuclease (TALEN) method, we found that neither maternal nor zygotic *stat3* functions are essential for the completion of embryogenesis. However, *stat3* mutants die during juvenile stages exhibiting scoliosis and excessive inflammation, warranting evaluation as a model for diseases such as scoliosis, cancer, and inflammatory diseases (e.g. HIES). Strikingly, rather than typical Wnt/PCP-based strong C&E defects, MZ*stat3* gastrulae manifested transient and mild extension defects in the axial and paraxial mesoderm. As the underlying cellular mechanism, we demonstrate that reduced cell number, due to decreased cell proliferation at blastula and gastrula stages and increased cell death during late gastrulation account for *stat3* mutant extension defects. We further show that the evolutionarily conserved Stat3 function of promoting cell cycle progression through upregulation of *cdc25a* expression is required for axis elongation during gastrulation.

## Results

### *stat3* mutants develop scoliosis, excessive inflammation, and fail to thrive

**Fig 1:**
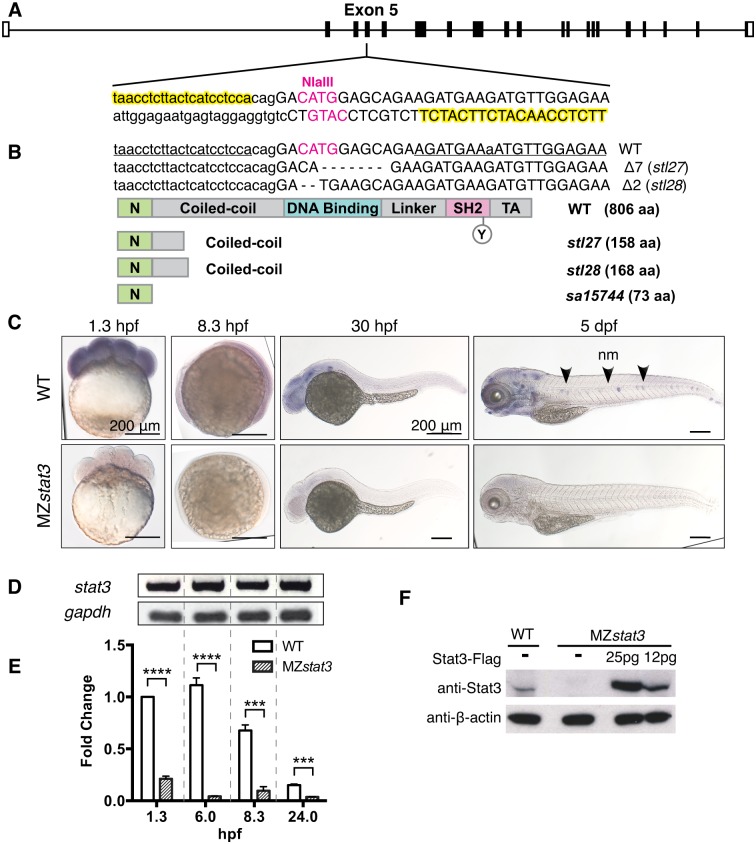
Zebrafish *stat3* mutants generated using TALEN method are strong/null alleles. (A) Design of TALEN pair targeting zebrafish *stat3* gene (yellow filled text). (B) Sequence alignments and schematics of Stat3 proteins encoded by *stl27*, *stl28* and *sa15744* alleles. (C) Expression patterns of *stat3* transcripts detected by WISH in WT and MZ*stat3* embryos (lateral views). Arrowhead, nm, neuromast. (D) RT-PCR analysis of *stat3* RNA levels in WT embryos at 1.3, 6, 8.3 and 24 hpf. *gapdh* was used as an internal control. (E) qRT-PCR analysis of *stat3* transcript levels in WT and MZ*stat3* embryos normalized to *gapdh*. (F) Western blot detecting total Stat3 in WT, MZ*stat3* and MZ*stat3* embryos overexpressing Flag-tagged Stat3 at 6 hpf. β-actin was used as a loading control. See also S1 Fig.

To extend functional studies of Stat3 in zebrafish, we generated mutations in the *stat3* gene using TALEN method (Fig 1A, see also Materials and methods). The *stat3*^*stl27*^ and *stat3*^*stl28*^ alleles contain 7- and 2-base pair deletions in Exon 5, respectively, resulting in frameshift and premature stop codons, encoding proteins predicted to lack almost all the critical functional domains of the Stat3 protein (Fig 1B).

**Fig 2:**
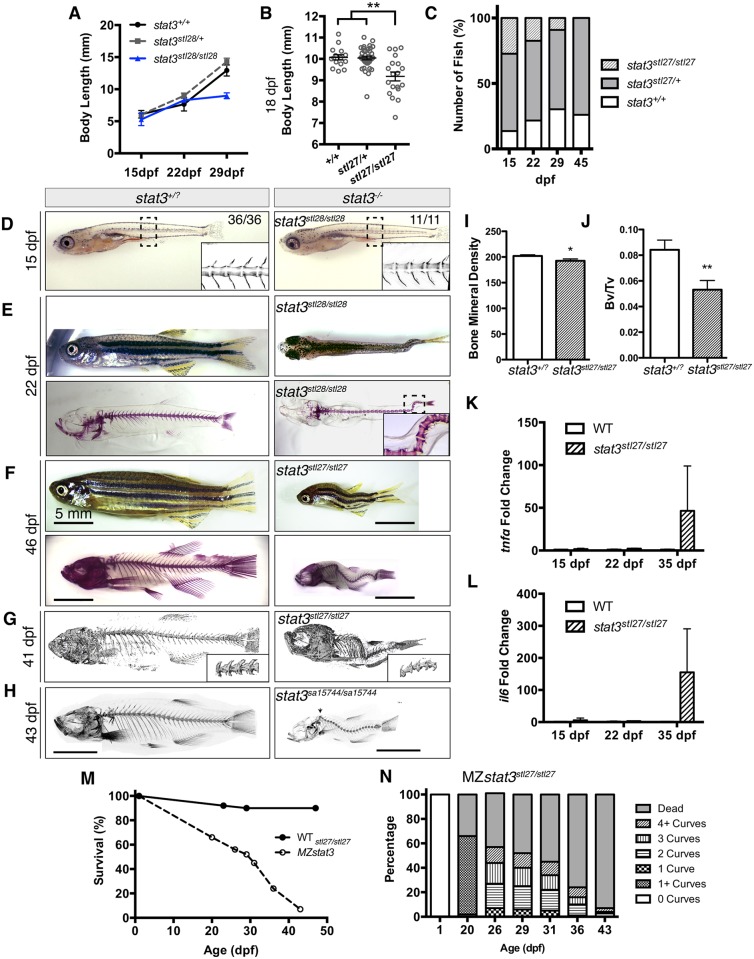
Zebrafish *stat3* mutants develop late-onset scoliosis and cannot survive to adulthood. (A) Growth curve of *stat3*^*stl28/stl28*^ fish and siblings. (B) Body length of *stat3*^*stl27/stl27*^ and *stat3*^*stl27/+*^ and WT siblings at 18 dpf. Different genotypes were kept in the same tank and fed exclusively with rotifers to diminish food competition. (C) Survival rate of *stat3*^*stl27/stl27*^, *stat3*^*stl27/+*^ fish and WT siblings. (D) Images of live animals and Alizarin stained skeletons showing WT and zygotic *stat3* mutant fish at 15 dpf (anterior to the left). Vertebral structures in the boxed region were revealed via Alizarin Red live staining and confocal imaging, shown in insets. (E) Images of live animals and Alizarin stained skeletons showing a WT and a zygotic *stat3*^*stl28/stl28*^ mutant fish with two curves near the caudal fin (inset) at 22 dpf. Anterior to the left. (F) Images of live animals and Alizarin stained skeletons showing a WT and a scoliotic *stat3*^*stl27/stl27*^ mutant fish at 46 dpf (anterior to the left). (G) μCT imaging showing body curvatures of WT sibling and *stat3*^*stl27/stl27*^ mutant at 41 dpf (anterior to the left). (H) μCT imaging showing body curvatures of WT sibling and *stat3*^*sa15744/sa15744*^ mutant at 43 dpf (anterior to the left, arrow denote bend in spine). (I-J) Quantification of bone mineral density (H) and bone/tissue volume ratio (Bv/Tv, I) of *stat3*^*stl27/stl27*^ (G). (K-L) Transcript levels of *tnfα* and *il6* in WT and *stat3*^*stl27/stl27*^ mutant animals detected by qRT-PCR at indicated developmental stages. (M-N) Survival rate and percentage of mutant fish with various numbers of body curves of MZ *stat3*^*stl27/stl27*^ mutants. *p<0.05, ***p<0.001, ****p<0.0001, error bars = SEM. See also S2 Fig.

Surprisingly, neither zygotic *stat3*^*stl27/stl27*^ nor *stat3*^*stl28/stl28*^ mutant embryos showed overt gastrulation defects described in the previous morpholino studies [12], and displayed normal morphology and notochord formation until 15 dpf (Fig 2A and 2D). During later larval stages, *stat3*^*stl28/stl28*^ (Fig 2A) and *stat3*^*stl27/stl27*^ (Fig 2B and 2F) mutant larvae appeared significantly smaller compared to siblings and manifested spinal curvatures in all dimensions (Fig 2E–2G). Scoliotic phenotypes could be discerned as early as 20 dpf (Fig 2E and 2N). Using Alizarin Red staining and micro-computed tomographic (micro-CT) imaging, we further detected three major categories of structural abnormalities in the vertebrae that contributed to spinal curvatures in *stat3* mutants (S2 Fig, S1–S3 Movies). In the first group, vertebrae with a straight vertebral body and perpendicular end plates display either gentle or sharp turns (S2B and S2C Fig). In the second group, abnormal vertebral morphology such as bent vertebral body and non-perpendicular end plates were observed in *stat3* mutant larvae (S2D and S2E Fig). Lastly, in two out of ten mutant larvae, we observed fractures and extra bony matrix (S2F–S2H Fig). Mutant animals were fragile and lethargic, and progressively died by 1.5 to 2 months of age (Fig 2C). Among progenies of *stat3*^*stl27/+*^ incross, mutants constituted 25% at 15 dpf; their proportion decreased over time, such that no mutants were detected at 45 dpf (Fig 2C). Micro-CT analyses revealed reduced bone mineral density (Fig 2I) and a nearly 40% decrease in the total bone volume in *stat3* juveniles (Fig 2J). Consistent with the roles of Stat3 in immune responses [6, 8], using qRT-PCR we observed a significant upregulation of transcripts encoding pro-inflammatory factors Tnfα and Interleukin (Il)-6 in *stat3*^*stl27/stl27*^ larvae and juveniles at 35 dpf (Fig 2K and 2L), subsequent to the manifestation of stunted growth and scoliotic phenotypes (Fig 2A, 2B and 2E). Given that both *stl27* and *stl28* alleles are predicted to cause frame shifts and produce similarly truncated proteins (Fig 1B), and that they resulted in similar growth defects, scoliotic phenotypes, and lethality (Fig 2), we conclude that they similarly disrupt *stat3* gene function. We also characterized, an additional *stat3*^*sa15744*^ allele, generated by chemical mutagenesis and high throughput sequencing, which is predicted to introduce a stop codon at amino acid 73 (Fig 1B) [34]. Fish homozygous for this allele exhibited phenotypes similar to the TALEN created alleles, including runting, scoliosis, gaping jaw, and larval stage lethality starting at about 19 dpf (Fig 2H). Given the similar phenotypes of the three TALEN *stat3* mutant alleles, we focused most of our studies on the *stat3*^*stl27*^ allele.

One hypothesis is that the growth defect observed in *stat3* homozygous mutants is due to failure to compete for food with their heterozygous and wild-type (WT) siblings in the same tank as a result of scoliosis and hence reduced mobility. To test this, we grew progeny of *stat3*^*stl27/+*^ heterozygotes on rotifer diet to reduce food competition with WT siblings (see Materials and methods). Larvae were fixed on 18 dpf, measured for body length, and genotyped. Homozygous zygotic *stat3* mutant larvae appeared to be 9% shorter than their siblings at 18 dpf (Fig 2B), indicating that growth retardation is a primary phenotype of *stat3* mutants. Interestingly, only one out of four scoliotic larvae was among the shortest; and in other experiments we also observed very short larvae of all genotypes without body curvatures (S2I Fig), arguing against a simple causal relationship between stunted growth and scoliosis phenotypes. Together these observations reveal that zygotic *stat3* function is dispensable for embryonic and early larval development in zebrafish. At juvenile stages loss of *stat3* function results in stunted growth, scoliosis, and inflammation that are not clearly causally related, and eventually death before sexual maturity.

### Maternal zygotic *stat3* gastrulae exhibit mild extension defect independent of PCP signaling

The lack of an overt embryogenesis defects in zygotic *stat3* mutants generated by heterozygous parents was surprising given previous reports of strong gastrulation defects in *stat3* morphants [12] and whole-mount *in situ* hybridization (WISH) studies that detected only zygotic expression of *stat3* in zebrafish embryos [35]. However, using WISH and RT-PCR we detected high levels of maternal *stat3* transcripts (Fig 1C and 1D). WISH further showed that *stat3* mRNA was expressed ubiquitously during blastula and gastrula stages and became enriched in the head and in neuromasts at 5 dpf (Fig 1C). To test whether maternal Stat3 function contributes to gastrulation, we generated maternal zygotic (MZ) *stat3*^*stl27/stl27*^ mutants by germline transplantations [29]. For simplicity, hereafter we use MZ*stat3*, Z*stat3*, and M*stat3* to refer to maternal zygotic (MZ*stat3*, from incrosses of WT fish harboring *stat3*^*stl27/stl27*^ germline), zygotic (Z*stat3*, from *stat3*^*stl27/+*^ incrosses), and maternal (M*stat3*^*stl27/+*^, from crosses of WT females harboring *stat3*^*stl27/stl27*^ germline with stat3^stl27/+^ or stat3^+/+^ males) mutant, respectively, unless indicated otherwise.

Unexpectedly, MZ*stat3* mutants progressed through embryogenesis and later exhibited phenotypes described above for Z*stat3* mutants with no observable changes in the onset, severity or survival (Fig 2C and 2M). Using qRT-PCR and four different primer pairs spanning regions at, upstream of, or downstream of the *stat3*^*stl27*^ deletion in all three *stat3* transcript sequences annotated in the Zv9 zebrafish genome assembly, we observed significant reduction of *stat3* transcripts in MZ*stat3* embryos (Fig 1E, S1A–S1C Fig). In agreement, Western blotting with an antibody against the C-terminus of zebrafish Stat3 failed to detect Stat3 protein in MZ*stat3* gastrulae (Fig 1F, S1D Fig, Materials and methods). Based on these results we conclude that *stl27* is a strong/null allele.

We first aimed to verify the scoliosis phenotype observed in zygotic *stat3* mutant larvae by growing 39 WT and 210 MZ*stat3* embryos in separate tanks. Whereas over 90% of WT larvae were alive at various time points of interest, survival of MZ*stat3* fish declined sharply over time, with 66% mutants alive at 20 dpf and only 7% at 43 dpf (Fig 2M). We also observed spinal curvatures in 100% of MZ*stat3* mutants from as early as 20 dpf, with majority of the larvae showing two, three, or four curves by 26 dpf (Fig 2N). Together, these results further confirmed that *stat3* function is essential for survival and spinal development and showed that the scoliosis phenotype and failure to thrive are completely penetrant.

**Fig 3:**
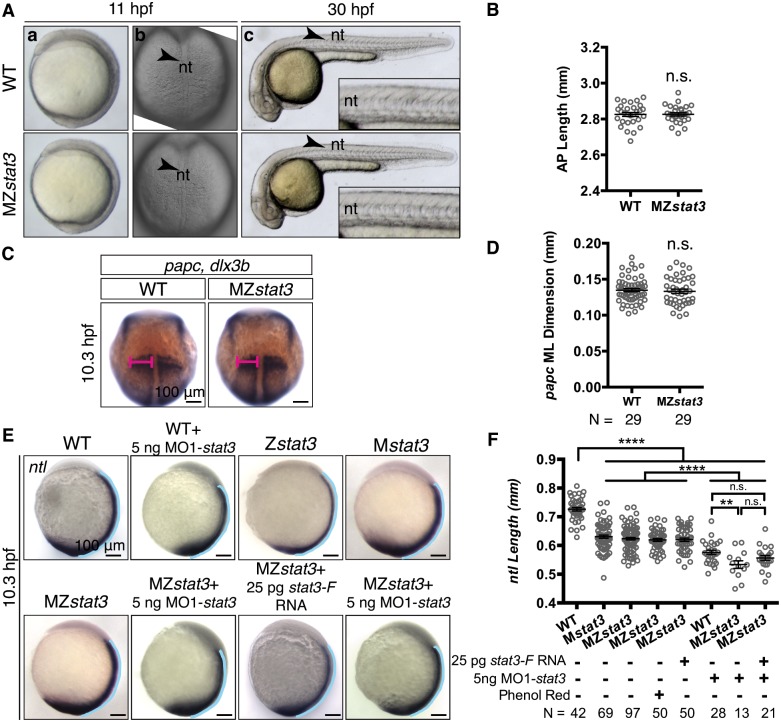
MZ*stat3* mutants exhibit transient and mild extension defects in axial mesoderm during gastrulation. (A) Live images of WT and MZ*stat3* embryos shown in lateral (a, c) and dorsal view (b). The insets in c show the part of notochord above yolk extension in 30 hpf embryos. Arrowhead, nt, notochord. (B) Morphometric analysis of AP axis extension of 30 hpf embryos shown in A(c). (C) *papc* in presomitic mesoderm and *dlx3b* marking neuroectoderm boundary in 1-somite stage WT and MZ*stat3* embryos (dorsal view). (D) Measurement of ML width of *papc* expression domain (pink in C). (E) Expression of *ntl* in notochord and tail in 1-somite stage WT, Z*stat3*, M*stat3*, MZ*stat3*, and MZ*stat3* embryos overexpressing Stat3-F, as well as WT and MZ*stat3* embryos injected with 5 ng MO1-*stat3* (lateral view). Phenol red was used as injection control. (F) Measurement of notochord length in embryos in E (blue lines in E). ****p<0.0001, n.s. = non-significant, error bars = SEM. See also S3 Fig.

Previous studies that employed antisense morpholino oligonucleotides (MO1-*stat3*) proposed a requirement of *stat3* in Wnt/PCP signaling and ML cell elongation essential for C&E gastrulation movements [13]. However, MZ*stat3* gastrulae did not exhibit severe C&E defects (Fig 3Aa and 3Ab) and showed normal AP body length by 30 hpf (Fig 3Ac and 3B). To detect any subtle morphogenetic defects, we visualized the nascent embryonic tissues that undergo dynamic C&E gastrulation movements using WISH [27, 36]. Whereas we failed to detect any defects in convergence of paraxial mesoderm assessed by the ML dimension of the *paraxial protocadherin* (*papc*)-expression domain (Fig 3C and 3D), we noticed 13.2% and 14.2% reduction compared to WT in the AP extension of the notochord marked by expression of *no tail* (*ntl*) in M*stat3* and MZ*stat3* mutants, respectively (Fig 3E and 3F). Phenotypic differences between morphants and mutants have been described for many zebrafish genes [37]. One possible mechanism involves genetic compensation, in which other genes alter their expression in the presence of a mutation, resulting in mild or no phenotypes [38]. To test whether such genetic compensation could explain the milder phenotypes of our MZ*stat3* mutants compared to *stat3* morphants [12], we injected MO1-*stat3* into both WT and MZ*stat3* one-celled embryos. If the lack of phenotype in MZ*stat3* embryos were due to genetic compensation, then they should show no additional C&E phenotypes when injected with morpholinos or even milder phenotype than WT zygotes injected with the same morpholino dose [38]. While injection of MO1-*stat3* into WT embryos resulted in dose-dependent body length reduction (S3A Fig), MZ*stat3* mutants showed comparable severe C&E phenotypes compared to WT gastrulae upon injection of the same MO1-*stat3* dose (Fig 3E and 3F). These results support the notion that the discrepancy between *stat3* morpholino- and mutation-induced phenotypes is likely due to off-target effects of MO1-*stat3* rather than to genetic compensation (see Discussion) [38].

**Fig 4:**
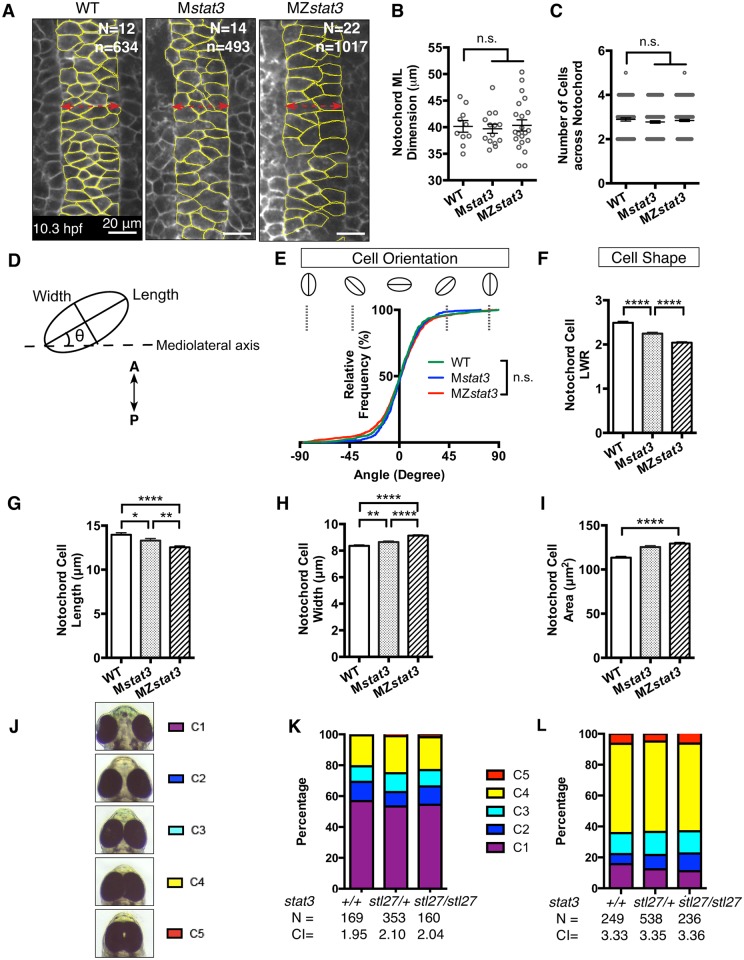
MZ*stat3* mutants show neither obvious cell polarity defects during C&E nor interaction with zebrafish PCP mutants. (A) Confocal image of dorsal mesoderm in WT, M*stat3*, and MZ*stat3* 1-somite stage embryos labeled with mGFP (anterior to the top). Cell shape and orientation of notochord cells outlined in yellow were analyzed as illustrated in D. (B) Measurement of notochord width at 1-somite stage. (C) Quantification of the number of cells across the ML notochord axis at 1-somite stage. (E) Cumulative distribution of notochord cell orientation in WT, M*stat3*, and MZ*stat3* embryos. (F) Cell shape analysis represented by length-to-width ratio (LWR). (G-I) Long axis (length, G), short axis (width, H) and average area (I) of in WT, M*stat3* and MZ*stat3* notochord cells. (J) A spectrum of eye separation phenotypes at 3 dpf with C1 representing WT eye spacing and C5 representing the most severe phenotype, cyclopia. Ventral view, anterior to the top. (K) Penetrance and expressivity of eye separation phenotypes of Z*tri*, Z*tri*;Z*stat3*^*stl27/+*^, and Z*tri*;Z*stat3* embryos. Eye separation phenotypes were also quantified by cyclopia index (CI) as previously described [36].(L) Penetrance and expressivity of eye separation phenotypes of MZ*slb*, MZ*slb*;Z*stat3*^*stl27/+*^, and MZ*slb*;Z*stat3* embryos. ****p<0.0001, n.s. = non-significant, error bars = SEM. See also S4 Fig.

During zebrafish gastrulation, ML intercalation of cells is the main driving force for C&E of the axial mesoderm tissue [39]. Hence, reduced AP length of axial mesoderm tissue in MZ*stat3* gastrulae (Fig 3E and 3F) could result from defective ML intercalation of cells. Against this notion, the notochord in WT, M*stat3* and MZ*stat3* mutant gastrulae at 1-somite stage (Fig 4A) showed comparable ML dimension (Fig 4B), an equivalent number of cells across the notochord in WT versus mutants (Fig 4C). Moreover, by mid-segmentation stage notochord in both M*stat3* and MZ*stat3* mutants converged to a single-cell column as observed in WT (S4 Fig).

We next asked whether ML cell elongation, the hallmark of Wnt/PCP signaling [25], was affected in *stat3*-deficient gastrulae. Our analyses revealed that at 1-somite stage the notochord cells in M*stat3* and MZ*stat3* mutants had a reduced length-to-width ratio (LWR) (2.0±0.0) compared to WT cells with LWR of 2.5±0.0 (Fig 4A, 4D and 4F–4H). However, contrasting a typical Wnt/PCP defect [27], the MZ*stat3* mutant notochord cells aligned normally their long axes with the ML embryonic axis (Fig 4E). Moreover, we noted that M*stat3* and MZ*stat3* mutant cells were 11.6% and 17.3% larger compared to their WT counterparts, respectively (Fig 4I). These results argue against Stat3 being a key regulator of Wnt/PCP signaling during zebrafish gastrulation, but also suggest a role of Stat3 in cell shape and size (see Discussion).

To query further whether Stat3 plays any role in Wnt/PCP signaling, we asked whether phenotypes of mutations disrupting Wnt/PCP components such as *trilobite(tri)/vangl2* [27] or *silberblick(slb)/wnt11* [26] could be exacerbated by simultaneous reduction of Stat3 function. A spectrum of eye separation phenotypes from partial to complete fusion of the eyes (Fig 4J), often associated with C&E defects, are commonly seen in both *tri* and *slb* embryos and are exacerbated in compound PCP mutants [36, 40]. However, we found that Z*stat3;*Z*tri*^*vu67/vu67*^ and Z*stat3*;MZ*slb*^*tz216/tz216*^ double mutants exhibited similar penetrance and expressivity of the eye separation defect compared to their single mutant siblings (Fig 4K and 4L). Together, our data provide genetic evidence for an essential role of Stat3 in normal AP axis extension during zebrafish gastrulation. Moreover, Stat3 regulates cell size and shape during gastrulation without significantly affecting Wnt/PCP signaling.

### Stat3 promotes cell proliferation but does not affect cell division orientation during early zebrafish embryogenesis

**Fig 5:**
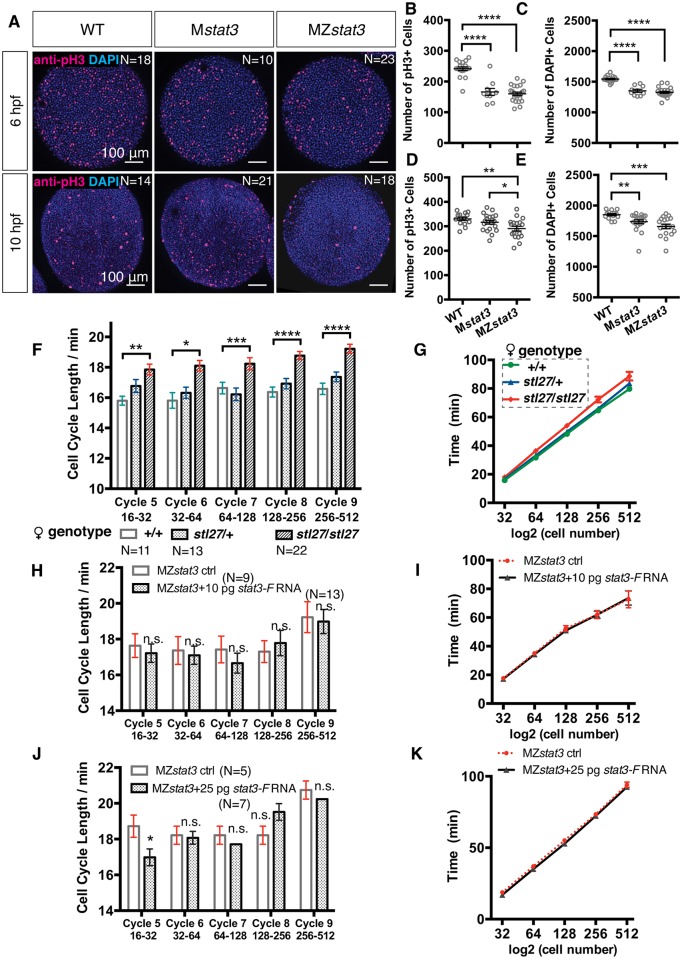
Stat3 promotes cell cycle progression during zebrafish embryogenesis. (A) Immunofluorescent anti-pH3 labeling of proliferating cells (red) and DAPI labeling all nuclei (blue) in WT, M*stat3*, and MZ*stat3* embryos at 6 hpf (animal view) and 10 hpf (dorsal view). (B, D) Quantification of mitotic cell number at 6 and 10 hpf. (C, E) Quantification of total cell number at 6 and 10 hpf. (F and G) Average length of each cell cycle (F) and timing of mitosis (G) from Cycle 5 to Cycle 9 in embryos from WT, *stat3*^*stl27/+*^, and *stat3*^*stl27/+*^ females. (H-K) Analyses of cell divisions from Cycle 5 to Cycle 9 in 10 pg (H and I) and 25 pg (J and K) *stat3-F* injected MZ*stat3* embryos. *p<0.05, **p<0.01, ***p<0.001, ****p<0.0001, n.s. = non-significant, error bars = SEM. See also S5 Fig.

In the above morphometric analyses, the enlarged cell size in MZ/M*stat3* mutant gastrulae stood out (Fig 4I). Consistent with reports of Stat3 promoting cell proliferation in many biological contexts [5], we detected 31.2% and 33.7% reduction in mitosis at early gastrulation (6 hpf) in M*stat3* and MZ*stat3* mutants compared to WT, respectively, as revealed by phosphorylated Histone H3 (pH3) immunostaining (Fig 5A and 5B). Total number of cells, determined by counting DAPI-stained nuclei, was also decreased by 12.5% and 14.1% in M*stat3* and MZ*stat3* mutants (Fig 5C), indicating comparable cell proliferation defects in these mutants at early gastrulation. By late gastrulation, however, M*stat3* gastrulae exhibited similar level of cell proliferation to that seen in WT (Fig 5D), whereas their total cell number continued to be reduced (Fig 5E), suggesting only a partial rescue of the cell proliferation defect by zygotic *stat3* expression. By contrast, both proliferation rate and total cell number in MZ*stat3* embryos remained low throughout gastrulation (Fig 5A, 5D and 5E). Together, these data indicate that Stat3 regulates cell proliferation during zebrafish embryogenesis. Moreover, one zygotic WT allele is not sufficient to compensate the cell number deficit caused by reduced cell proliferation in maternal *stat3* mutants, revealing a crucial role of maternal Stat3.

To investigate the maternal Stat3 function in cell proliferation, we analyzed the rapid cleavages prior to MBT that depend exclusively on maternally deposited proteins and RNAs [41], via *in vivo* confocal time-lapse imaging of embryos with Histone2B-RFP (H2B-RFP)-labeled nuclei (Materials and methods). Whereas WT blastomeres divided every 15.8 ~ 16.6 min from Cycle 5 (from 16 to 32 cells) to Cycle 9 (from 256 to 512 cells) (Fig 5F, S5 Fig, S4 Movie) consistent with previous reports [18], pre-MBT cycles ranged from 17.6 min to 19.2 min in embryos lacking maternal *stat3* function (Fig 5F, S5 Movie), a nearly 13% increase. Cumulatively, M*stat3* mutants took significantly longer to complete five pre-MBT cell cycles compared to progeny of WT and heterozygous females (Fig 5F and 5G). Interestingly, embryos obtained from heterozygous females exhibited normal length of the early cleavage cycles (Fig 5F), indicating that one WT *stat3* allele in the female germline is sufficient to ensure in the progeny normal embryonic cleavages before the initiation of zygotic transcription.

**Fig 6:**
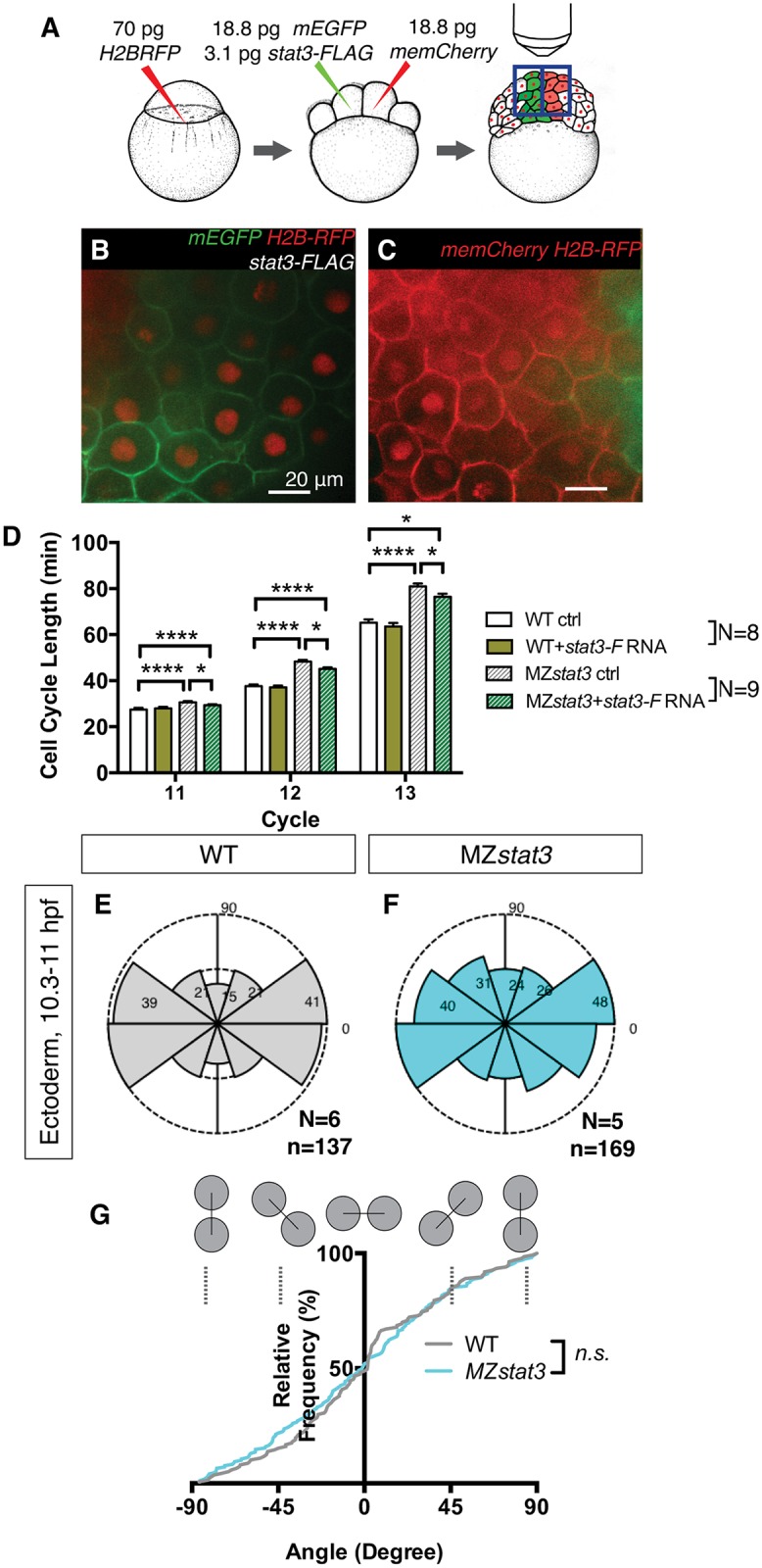
Stat3 promotes post-MBT cell divisions but does not regulate cell division orientation during zebrafish embryogenesis. (A) Experimental design for post-MBT cell cycle analyses. Embryos labeled ubiquitously with histone2B-RFP (H2B-RFP) were mosaically injected with *memCherry* or *mGFP* + *stat3-F* mRNA at 8-cell stage for lineage tracing. Labeled clones (B and C) within the same embryo were monitored using confocal time-lapse microscopy (See also Materials and methods). (D) Analyses of cell cycle lengths for Cycle 11–13 in WT, WT overexpressing Stat3-F, MZ*stat3*, and MZ*stat3* overexpressing Stat3-F embryos. (E-G) Cell division orientation in dorsal neuroectoderm in WT (E) and MZ*stat3* (F) embryos during 1–3 somite stages, with 0 degrees representing mediolaterally aligned cell divisions and 90 degrees representing anteroposteriorly aligned cell divisions, quantified in G. *p<0.05, **p<0.01, ****p<0.0001, n.s. = non-significant, error bars = SEM. See also S7 Fig.

We next asked whether Stat3 is required for post-MBT cell divisions. Our manual lineage tracing of individual blastomeres for the duration of five post-MBT divisions (Fig 6A–6C, Materials and methods) revealed that cell cycles gradually lengthened from MBT onward in both WT and MZ*stat3* embryos, consistent with previous observations [19]. Furthermore, cycles 10 through 13 were significantly longer in MZ*stat3* than in WT embryos (Fig 6D), demonstrating Stat3 was required for post-MBT cell cycle progression. Together, these results establish a key role of Stat3 in promoting cell proliferation throughout early embryogenesis.

Alignment of cell division with the AP embryonic axis during early gastrulation has been shown to be regulated by Wnt/PCP pathway, although it remains unresolved whether polarized cell division contributes to axis extension [32, 33]. Oriented cell division during late gastrulation and early segmentation is essential for morphogenesis such as neural rod midline formation [32, 33]. To test Stat3’s role in cell division orientation, we analyzed time-lapse movies of WT and MZ*stat3* embryos at late gastrulation and early segmentation stages (1–3 somite stage). Cell divisions of dorsal neuroectodermal cells were mediolaterally aligned in both WT and mutant gastrulae (Fig 6E–6G). Together these results indicate a requirement for both maternal and zygotic Stat3 function in promoting cell divisions before and after MBT, but argue against a significant role of Stat3 in cell division orientation during early zebrafish embryogenesis.

### *stat3* deficiency increases apoptosis

**Fig 7:**
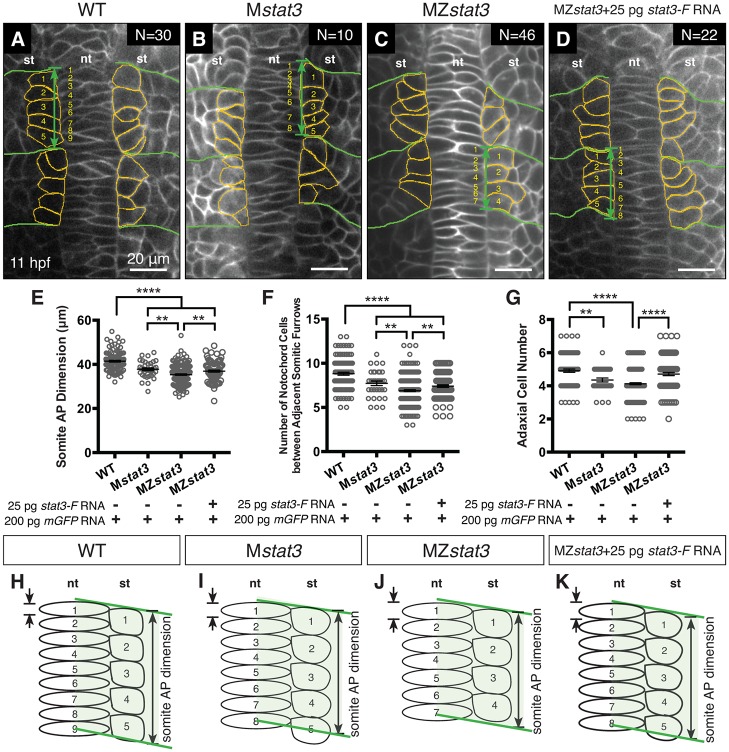
Cell number reduction correlates with axis extension defects in *stat3* mutant embryos. (A-D) Confocal image of dorsal mesoderm expressing mGFP in WT (A), M*stat3* (B), MZ*stat3* (C) embryos, and MZ*stat3* embryos overexpressing Stat3-F (D) at 11 hpf (anterior to the top). Somite boundaries are outlined in green. Adaxial cells are outlined in orange. Green arrows show AP dimension of each somite. Adaxial cells and notochord cells aligning along the AP axis are numbered in yellow. (E-G) Quantification of the average somite AP dimension (E), number of notochord cells (F) and number of adaxial cells (G) between adjacent furrows of the first two somites. (H-K) Schema of AP extension of the notochord and presomitic mesoderm. st, somite; nt, notochord. **p<0.01, ****p<0.0001, error bars = SEM.

Stat3 suppresses apoptosis in various biological contexts through transcriptional activation of anti-apoptotic genes [5]. During zebrafish embryogenesis, apoptosis can be detected from late gastrulation/early segmentation stages [42]. Terminal deoxynucleotidyl transferase mediated dUTP Nick End Labeling (TUNEL) assay did not detect apoptotic cells in WT or MZ*stat3* embryos at midgastrulation (60% epiboly or 6.5 hpf and 80% epiboly or 8.3hpf), but revealed a nearly 70% increase in the number of apoptotic cells in MZ*stat3* embryos compared to WT at both late gastrulation (10 hpf; S6A–S6C Fig) and early somitogenesis (11hpf; S6D–S6F Fig). Using qRT-PCR, we further detected substantial downregulation of *birc5a*, the zebrafish homolog of known anti-apoptotic target of Stat3, *Survivin* [43], in MZ*stat3* embryos (S6G Fig), whereas expression of *bcl2a* was not significantly altered (S6H Fig). Together, these data indicate that Stat3 suppresses apoptosis in zebrafish embryos likely through activating anti-apoptotic genes such as *birc5a/survivin*. Moreover, elevated apoptosis may further decrease the number of cells in MZ*stat3* mutants at late gastrulation (Fig 5E) and thus contribute to the axis extension defect (Figs 3E and 7).

### Reduced cell proliferation impairs axis extension

During ML cell intercalation in vertebrate gastrulae cells move medially or laterally to separate anterior and posterior neighbors and align one after another anteroposteriorly, simultaneously producing AP extension and ML convergence [24]. Confocal imaging at 1 somite stage revealed normal width and 2–4 rows of cells in the notochords of both MZ*stat3* and WT gastrulae (Fig 4A–4C). At the 3-somite stage, we observed two rows of ML elongated axial mesodermal cells in the notochords of both MZ*stat3* and WT gastrulae (Fig 7A–7C), arguing against defective ML intercalation. Rather, we reasoned that a shorter notochord in MZ*stat3* gastrulae results from fewer cells that participate in ML intercalation, which drives the anteroposterior extension of dorsal mesoderm, due to decreased cell proliferation (Fig 5A–5E) and increased apoptosis (S6 Fig). To test this, we analyzed the dimensions and numbers of cells in one optical section of the notochord and adjacent first three somites in 3-somite stage embryos (see Materials and methods). Within each somite, flanking the notochord are the adaxial cells that later give rise to slow muscles [44]. We found that somites were 8.9% and 14.7% shorter in AP dimension in M*stat3* and MZ*stat3* mutants compared to WT, respectively (Fig 7E). Correlated with the AP extension defect, somites exhibited 11.4% (M*stat3*) and 16.1% (MZ*stat3*) fewer adaxial cells compared to WT somites, which contained 4.9 ± 0.1 adaxial cells. Likewise, the adjacent notochord tissue contained 12.2% (M*stat3*) and 21.7% (MZ*stat3*) fewer cells than in WT embryos, in which the corresponding notochord fragment featured 8.8 ± 0.2 cells (Fig 7F and 7G). These results support the model whereby reduction of cell number in M*stat3* and MZ*stat3* mutants contributes to morphogenetic defects in extension of axial and presomitic mesoderm (Fig 7H–7J), despite enlarged cell size (Fig 4I).

**Fig 8:**
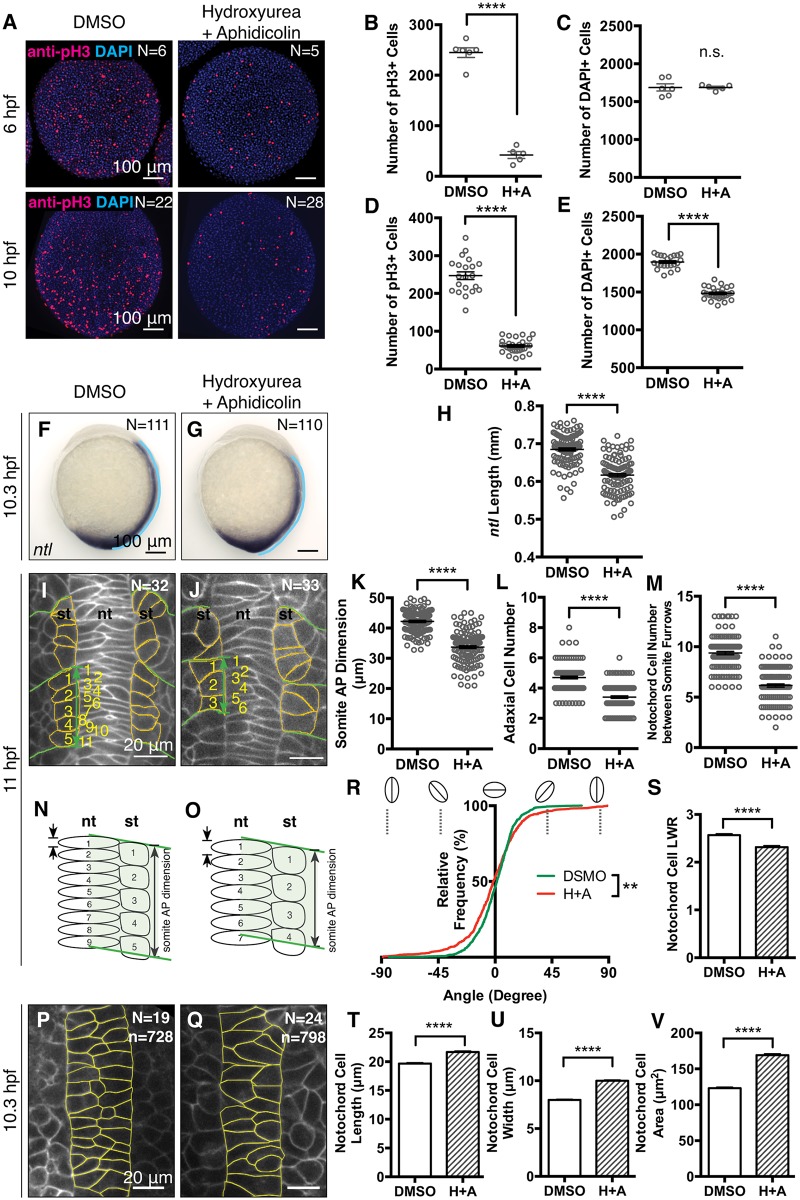
Inhibition of cell proliferation using hydroxyurea and aphidicolin leads to axis extension defects in zebrafish gastrulae. (A) Immunofluorescent anti-pH3 labeling of proliferating cells (red) and total nuclei labeled with DAPI labeling (blue) in DMSO-treated control embryos and hydroxyurea+aphidicolin (H+A)-treated embryos at 6 hpf (animal view) and 10 hpf (dorsal view, anterior to the top). (B-E) Quantification of pH3+ cells (B and D) and DAPI+ cells (C and E) in A. (F and G) Expression of *ntl* at 1-somite stage (lateral view, anterior to the top). (H) Quantification of notochord length (blue lines in F and G). (I and J) Confocal image of dorsal mesoderm in 3-somite stage embryos expressing mGFP with somite AP dimension illustrated with green arrow, somitic boundaries outlined in green, adaxial cells outlined in orange, adaxial cells and notochord cells between adjacent somitic boundaries numbered in yellow (dorsal view, anterior to the top). (K-M) Quantification of somite AP dimension (K), numbers of adaxial cells (L) and notochord cells (M) in I and J. (N and O) Schema of AP extension of the notochord and presomitic mesoderm. st, somite; nt, notochord. (P-V) Dorsal view showing cells labeled with mGFP in DMSO-treated (P) and drug-treated (Q) embryos at 1-somite stage (anterior to the top). Analyses of notochord cells’ orientation (R), shape (S), long axis (length, T), short axis (width, U) and size (V). ****p<0.0001, n.s. = non-significant, error bars = SEM.

To test this model further, we asked whether chemical inhibition of cell proliferation in WT gastrulae could phenocopy the MZ*stat3* axis extension defect. Inhibiting proliferation in WT embryos with 150 μM aphidicolin and 20 mM hydroxyurea from early shield stage (5.7 hpf, Fig 8A–8D) [31], resulted in 22% reduction of total cell number by late gastrulation (10 hpf, Fig 8C–8E), similar to that in MZ*stat3* mutants (Fig 5E). Moreover, compared to DMSO-treated controls, *ntl*-expressing notochord was 10% shorter in drug-treated embryos at 1-somite stage (Fig 8F–8H). At 3-somite stage, somites were 20% shorter in AP dimension and had 27.5% fewer adaxial cells (Fig 8I–8L), with the corresponding notochord fragment possessing 34.3% fewer cells (Fig 8M). Together, these results indicate that drug inhibition of cell proliferation phenocopied both proliferation and morphogenetic defects caused by loss of *stat3* function, supporting the model whereby reduced cell number in MZ*stat3* embryos due to proliferation defect could account for impaired extension. Hence, cell proliferation is required for normal axis extension during zebrafish gastrulation.

We next asked if the altered cell shape observed in MZ*stat3* gastrulae was due to increased cell size (Fig 4F–4I). Similarly to MZ*stat3* mutants, drug treatment increased notochord cell size (Fig 8P, 8Q and 8V) with only slight change in their ML alignment (Fig 8R). However, the enlarged cells in drug-treated embryos featured greater long and short axes, and their cell elongation was only slightly reduced (LWR = 2.3) compared to that of cells from DMSO-treated controls (LWR = 2.6, Fig 8S, 8T and 8U). This contrasted the cell shape defect of MZ*stat3* mutants, where enlarged cells showed diminished long but increased short axes, and therefore strongly reduced LWR (2.0±0.0, Fig 4F–4H). These results argue against the cell size increase alone causing the cell shape defect in MZ*stat3* gastrulae.

### Stat3 overexpression partially rescues post- but not pre-MBT cell proliferation defect in MZ*stat3* mutants

We next asked whether restoring Stat3 expression could rescue proliferation and/or axis extension phenotypes in *stat3*-deficient embryos by injecting into 1-celled zygotes synthetic RNA encoding zebrafish Stat3 with FLAG tag at the C-terminus (Stat3-F). However, injection of neither 10 pg nor 25 pg *stat3-F* RNA altered the lengths of the cell cycles preceding initiation of zygotic transcription in MZ*stat3* mutants (Fig 5H–5K). For post-MBT cell divisions, we mosaically overexpressed Stat3-F in MZ*stat3* embryos labeled ubiquitously with H2B-RFP (Fig 6A–6C; Materials and methods). Notably, in MZ*stat3* cells overexpressing Stat3-F, Cycles 11~13 were shorter compared to those in uninjected mutant cells, but still longer than WT cycles (Fig 6D), indicating a partial rescue of post-MBT cell division defect. Interestingly, we did not observe significant changes of cell cycle length in WT cells overexpressing Stat3-F (Fig 6D). We also verified in WT and MZ*stat3* embryos that post-MBT cell cycle lengths were not altered in cells injected with RNA encoding fluorescent proteins (S7A and S7B Fig).

Although injection at 1-cell stage of 25 pg *stat3-F* RNA did not significantly rescue reduced axis extension in MZ*stat3* mutants as assayed by *ntl* WISH at 1-somite stage (Fig 3E and 3F), it partially rescued the somite AP extension and notochord cell number phenotypes and completely normalized adaxial cell number at 3-somite stage (Fig 7D–7G and 7K). Hence, restoring Stat3 expression in MZ*stat3* mutants could partially rescue phenotypes caused by defects in post-MBT processes such as post-MBT cell divisions; but failed to rescue the deficits caused by pre-MBT defects. These observations corroborate the critical function of maternally contributed Stat3, and imply that the role of Stat3 in cell proliferation during zebrafish embryogenesis is transcription-dependent.

### Stat3 regulates cell proliferation and axis extension by promoting cdc25a expression

**Fig 9:**
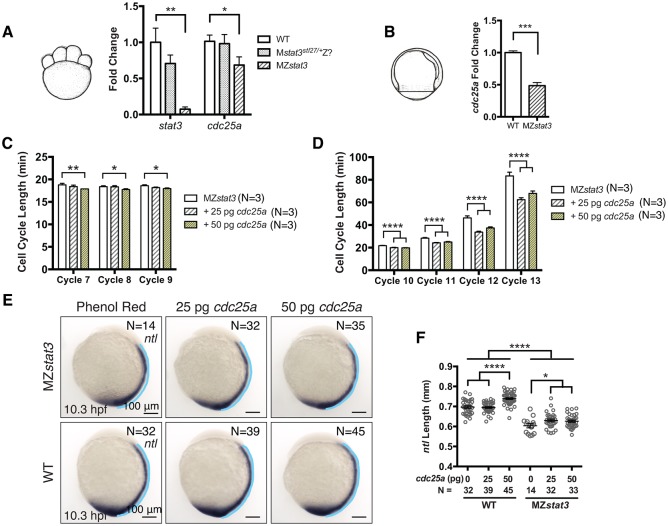
Stat3 promotes cell cycle progression during zebrafish embryogenesis via upregulation of *cdc25a* expression. (A) qRT-PCT analysis of *stat3* and *cdc25a* transcript levels at 1.5 hpf. All results shown were normalized to *gapdh*. (B) qRT-PCR analysis of *cdc25a* transcript levels at 8.3 hpf. (C and D) Pre-MBT (C) and post-MBT (D) cell cycle length analyses in 1-somite stage MZ*stat3* embryos injected with 25 or 50 pg *cdc25a* RNA. Phenol Red was used as injection control. (E and F) *ntl* expression in 1-somite stage MZ*stat3* and WT gastrulae misexpressing Cdc25a (lateral view, anterior to the top). Notochord length (blue line) was quantified in F. *p<0.05, **p<0.01, ***p<0.001, ****p<0.0001, error bars = SEM. See also S9 Fig.

We next wished to define the molecular mechanism through which Stat3 regulates cell proliferation. Stat3 is known to regulate transcription of many cell cycle regulators [5]. Accordingly, qRT-PCR revealed significant downregulation of *cdc25a* RNA in MZ*stat3* mutants during cleavage and gastrula stages (Fig 9A and 9B). In addition, expression of genes encoding Cyclins, such as *ccna2*, *ccnb1*, and *ccnb2*, was slightly but not statistically significantly reduced with exception of *cyclinD1* (S8A Fig).

Cdc25a has a conserved role in promoting mitotic entry in early animal development [19–21]. Hence, we asked if restoring *cdc25a* expression could suppress cell cycle and axis extension defects in MZ*stat3* mutants. Accordingly, we observed shortening of pre-MBT cycles (Cycle 7~9) in mutant embryos injected with 50 pg *cdc25a* RNA (but not with 25 pg *cdc25* RNA), with cells dividing every 17.7~17.9 min compared to 18.4~18.8 min in control MZ*stat3* embryos (Fig 9C). Further, injection of either 25 pg or 50 pg *cdc25a* RNA fully suppressed post-MBT cell cycle phenotype (Fig 9D), and partially suppressed notochord extension defect in MZ*stat3* gastrulae (Fig 9E and 9F). Notably, injection of 50 pg *cdc25a* RNA also resulted in excess notochord extension in WT gastrulae (Fig 9E and 9F). Injection of 25 to 50 pg *cdc25a* RNA significantly reduced size and width of the notochord cells in 1-somite stage WT gastrulae without affecting cell body orientation (S9A–S9H Fig). Although not statistically significant, there appeared to be more notochord cells lined up between adjacent somitic furrows in *cdc25a*-overexpressing WT embryos at 3-somite stage (S9M Fig), opposite to what we observed in MZ*stat3* or cell division inhibitor-treated embryos (Figs 4 and 8). Based on these results we propose that Stat3 regulates cell proliferation in zebrafish embryogenesis in part by regulating c*dc25a* expression, and that Stat3/Cdc25a-dependent cell proliferation promotes axis extension during gastrulation.

## Discussion

**Fig 10:**
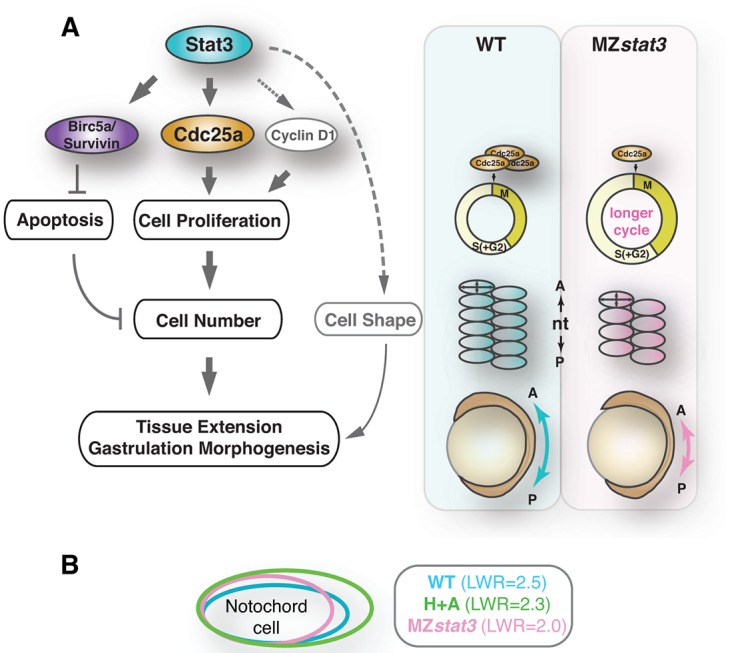
Model of Stat3/Cdc25a signaling regulating morphogenesis during zebrafish embryogenesis. (A) Model of Stat3 regulating morphogenesis during zebrafish embryogenesis by promoting Cdc25 expression and cell proliferation. Stat3, both maternal and zygotic, upregulates Cdc25a expression to promote cell division throughout early development of zebrafish embryos. Loss of *stat3* leads to reduced Cdc25a levels, longer cell cycle both pre- and post-MBT, and hence overall reduction of cell number. Consequently, fewer but bigger dorsal mesoderm cells participate in cell intercalations to align along the AP axis during gastrulation, causing extension defects. (B) Illustration of notochord cell shape in WT (blue), MZ*stat3* (pink) and drug-treated (green) 1-somite stage embryos.

Stat3 was reported to control C&E movements during zebrafish gastrulation partly through promoting Wnt/PCP signaling [12, 13]. Our analyses of the newly generated zebrafish *stat3* null mutants do not support a requirement of Stat3 in convergence movements or in Wnt/PCP signaling. Instead, we propose an alternative model in which maternal and zygotic Stat3 function promotes axis extension by ensuring sufficient number of cells are engaged in C&E movements, through regulation of Cdc25a-dependent cell proliferation and cell survival (Fig 10A, S6 Fig). Further, the scoliosis phenotype of juvenile zebrafish *stat3* mutants provides possible new opportunities to study scoliosis in a model organism.

### The zebrafish *stat3* mutations

The frame-shift mutations we generated in the zebrafish *stat3* gene predicted to encode truncated proteins lacking all functional domains are likely strong/null mutations as evidenced by significant reduction of *stat3* transcripts and undetectable level of Stat3 protein in the MZ*stat3*^*stl27/stl27*^ mutants (Fig 1C, 1E and 1F). In contrast to previous findings [35], we show that *stat3* is also maternally expressed. Nevertheless, both zygotic and MZ *stat3* mutants complete embryogenesis (Fig 1C), indicating that *stat3* is not essential for embryonic development in zebrafish.

Contrasting the *stat3* morpholino studies [12], MZ*stat3* gastrulae exhibited normal convergence and only mild and transient axis extension defects largely due to reduced cell proliferation and increased apoptosis. Post-MBT cell proliferation and axis extension defects could be partially rescued by Stat3 overexpression, which further supports that the phenotypes caused by *stl27* and *stl28* alleles are specific to loss of *stat3* function (Figs 5 and 6). The elevated apoptosis at late gastrulation and early segmentation (S6 Fig) exacerbates cell deficit in MZ*stat3* mutants and likely contributes to the axis extension defect. However, cell death is unlikely a major contributor to the axis extension defect in MZ*stat3* embryos, as C&E of the axial mesodermal tissue starts as early as 7.3 hpf [39], but apoptosis only becomes detectable from 10 hpf in both WT and MZ*stat3* embryos (S6 Fig). Therefore, apoptosis is less likely to have a significant impact on axis extension at the time of our analyses (1-somite stage, or 10.3 hpf). In addition, there are nearly ca. 100 and 50 fewer cells undergoing mitosis as revealed by pH3 staining at 6 and 10 hpf (Fig 5B and 5D), respectively, compared to only ca. 30 more cells undergoing apoptosis in MZ*stat3* mutant embryos at 10 hpf (S6C Fig). Together, whereas increased cell death likely contributes to the axial extension defect in MZ*stat3* gastrulae, we consider reduced cell proliferation as the primary cellular basis for the axis extension defect in M and MZ*stat3* mutants.

Neither cell proliferation defect nor apoptosis was reported for *stat3* morphant gastrulae. Injection of 10 pg *stat3* morpholino as reported in Yamashita et al [12] or 5 pg morpholino as tested in our study, led to significant developmental delay, increased apoptosis, and later necrosis in the head region at 1 dpf; with morphants dying between 1 and 5 dpf [12]. The discrepancy between the reported *stat3* morphant and *stat3* mutant phenotypes described here resonates with recent reports of poor correlation between morpholino-induced and mutant phenotypes in zebrafish [37]. There could be several causes for this phenotypic discrepancy between *stat3* morphant and mutant phenotypes. Firstly, different strategies may result in different degrees of functional *stat3* inactivation. Whereas both MO1-*stat3* [12] and mutations were able to deplete Stat3 protein during gastrulation (Fig 1, S1 Fig), only inactivation of *stat3* in the female germline (by transplanting *stat3* mutant germline into WT blastulae) but not injections of MO1-*stat3* into fertilized zygotes, can inactivate *stat3* function during oogenesis. An essential function of Stat3 in the pre-MBT cleavages is underscored by the observation that injections of RNA encoding Stat3-F into MZ*stat3* one-celled zygote failed to normalize pre-MBT cell divisions (Fig 5H–5K), while being able to partially rescue the post-MBT cell division defects (Fig 6A–6D). We propose that Stat3 promotes the transcription of *cdc25a* during oogenesis to ensure normal early cleavages before MBT. This proposed mechanism can explain why the cell proliferation defect was clear in MZ*stat3* mutants (Fig 5) but was not reported in the morphants [12], as translation-blocking MO1-*stat3* injected after fertilization could not interfere with the function of *stat3* during oogenesis. Secondly, a recent study reported that phenotypic differences between zebrafish morpholino knockdown and mutants could be explained by genetic compensation induced by deleterious mutations with transcriptome being fine-tuned for adaptation [38]. For example, in the *egfl* case, where such genetic compensation was observed in the mutants, *egfl* mutants appeared less sensitive than WT to *egfl* morpholino injections due to upregulation of downstream genes in the mutants compensating for loss of the *egfl* function. However, the fact that MZ*stat3* mutant embryos were not less sensitive than WT to MO1-*stat3* injection argues against such genetic compensation accounting for the mild gastrulation phenotypes observed in MZ*stat3* mutants (Fig 3E and 3F) [38]. Therefore, the phenotypic discrepancy between *stat3* morphants and mutants is likely due to off-target effects of MO1-*stat3*. Further, the zebrafish *stat3* mutants described here afford a reliable tool to verify and investigate other proposed functions of Stat3, such as in retina regeneration [17].

### Stat3/Cdc25a axis regulates cell proliferation in development

We have established a requirement of Stat3, particularly maternal Stat3, in both pre- and post-MBT cell proliferation during zebrafish embryogenesis. In the absence of both maternal and zygotic Stat3 functions, cell cycles are longer (Figs 5 and 6). Moreover, *stat3* mutants exhibited severe growth defects from late larval stage (Fig 2A and 2B), suggesting a continuous requirement of Stat3 for cell proliferation throughout zebrafish development. Several observations argue that the cell proliferation defects and reduced axis extension in MZ*stat3* mutants are not associated with or caused by developmental delay. First, a number of key morphogenetic processes occurred in MZ*stat3* mutants contemporaneously with such events in WT embryos. For example, the dorsal embryonic shield formed on time and epiboly progression was not delayed in MZ*stat3* embryos despite elongated cell cycles (Fig 3A). Convergence movements of lateral mesodermal cells, which are initiated at midgastrulation and narrow mesoderm mediolaterally, occurred normally in MZ*stat3* mutants (Fig 3C and 3D). In addition, segmentation is considered a key staging index in zebrafish and other vertebrates, and MZ*stat3* mutants exhibited the same number of somites as time-matched WT embryos (Figs 3Ab and 7A–7C). Second, tissue-specific gene expression occurred at equivalent times in MZ*stat3* mutants compared to time-matched WT embryos. For example, zygotic gene expression of *bozozok/dharma* at 4 hpf and *floatinghead* at 6 hpf occurred on time in MZ*stat3* embryos (S10 Fig) [45]. Further the mediolateral dimension of the floating head expression domain, which is shaped by dynamic anterior migration and convergence movements was not significantly different than in control WT embryos. In addition, MZ*stat3* mutants at 10.3 hpf exhibited equivalent to WT expression of *papc* presomitic and *dlx3b* ectodermal marker genes (Fig 3C). All these lines of evidence indicate that the axial extension defects of MZ*stat3* mutant gastrulae do not reflect developmental delay, but rather a specific morphogenetic defect.

Therefore, our studies support the notion that when the cell cycle length and consequently cell division number are uncoupled from the normal developmental schedule, this leads to morphogenetic defects like axis shortening. Such morphogenetic defects, even if transient, can impact inductive interactions between tissues. For example, we previously reported that C&E movements regulate the number of adaxial cells, slow muscle fiber precursors that are specified during gastrulation, by determining the size of the interface between the inductive axial and target presomitic tissues [46]. Given that MZ*stat3* mutants exhibit smaller number of larger adaxial cells during segmentation (Fig 7C and 7F), it will be interesting to investigate development of slow muscle fibers in these mutants.

Cell cycle control is a conserved role of Stat3 in animal development and cancer [5]. Our data support a model whereby Stat3 promotes cell divisions during zebrafish embryogenesis in part through transcriptional activation of Cdc25a, as in MZ*stat3* embryos *cdc25a* transcripts were significantly downregulated (Fig 9A and 9B). Moreover, ectopic *cdc25a* RNA suppressed both pre-MBT and post-MBT cell cycle phenotypes (Fig 9C and 9D) while providing ectopic *stat3* RNA from 1-cell stage rescued only post-MBT but not pre-MBT cell cycle defect in MZ*stat3* mutants (Figs 5 and 6). A key regulator of G1-S and G2-M transitions, CDC25a is overexpressed in human cancers driving abnormal cell proliferation downstream of multiple signaling pathways including STAT3 [47]. In HepG2 carcinoma cells, for example, STAT3 binds directly to *CDC25a* promoter and activates its expression [48]. Cdc25a is also a conserved regulator of cell divisions during embryogenesis from *Drosophila* to *Xenopus*, where pre-MBT mitotic entry is propelled by Cdc25a synthesized from maternal RNAs through activation of Cyclin B/Cdk1 complexes [20–23]. Cdc25a activity is continuously required after MBT, as cells are arrested in G2 in the *Drosophila cdc25*/*string* mutant [49] and zebrafish *cdc25a*/*standstill* mutant [50]. Whereas it was unclear how *cdc25a* expression is activated in these early embryos, our studies point to Stat3 as a regulator of *cdc25a* during zebrafish development, paralleling this role in cancer [48]. Furthermore, Stat3/Cdc25a pathway may be conserved in mammalian embryogenesis. First, *Stat3* and *Cdc25a* knockout mice both die by early gastrulation; when cultured, both *Stat3-/-* and *Cdc25a-/-* mouse blastocysts exhibit growth defects [11, 51]. Second, *STAT3* mutant homozygotes have never been reported in human, while spontaneous dominant-negative *STAT3* mutations have been linked to autosomal dominant HIES [6], suggesting that *STAT3* inactivation causes embryonic lethality in humans. Hence, the Stat3/Cdc25a pathway may serve as a universal mechanism regulating cell proliferation during animal embryogenesis.

However, our results imply other players downstream of Stat3 are involved. First, we detected downregulation of other cell cycle-regulating genes in MZ*stat3* embryos including *ccnd1* encoding Cyclin D1 (S8A Fig). Second, Stat3 overexpression failed to normalize *cdc25a* transcript level in whole MZ*stat3* gastrulae (S8B Fig). Given the tissue-specific requirement of Stat3 we observed (Fig 7), Stat3-dependent *cdc25a* activation may only occur within certain tissues and would be difficult to detect in the context of a whole embryo.

### Cell proliferation promotes axis extension

Cell proliferation has been generally considered dispensable or even prohibitive for gastrulation movements and morphogenesis. For example, cell shape changes and ventral furrow formation in *Drosophila* require the inhibition of ventral cell proliferation through String/Cdc25 inhibitors Tribbles and Frühstart [52]. In *Xenopus*, increased cell proliferation induced by inhibition of Wee2, a Cdk negative regulator, impaired C&E in the paraxial mesoderm [28]. Conversely, zebrafish gastrulae still achieved relatively normal AP axis extension when mitosis was chemically inhibited [32], although morphometric analyses have not been carried out. Indeed, a mathematical modeling of zebrafish gastrulation indicated that directed cell migration and polarized cell intercalation, the motile cell behaviors mediated by Wnt/PCP pathway, are largely sufficient to account for the morphogenesis of paraxial mesoderm given that cell divisions are very infrequent in the course of this process, although a minor role of cell proliferation could not be excluded [53].

We present evidence in support of a small but significant contribution of cell proliferation to zebrafish gastrulation by showing that cell proliferation during blastula and/or gastrula stages promotes and is required for AP extension of both the axial and paraxial mesoderm. The most compelling corroboration of our MZ*stat3* mutant analyses comes from pharmacological experiments where we inhibited mitosis in WT zebrafish embryos during gastrulation with hydroxyurea and aphidicolin [32]. Drug treatment during gastrulation recapitulated both proliferation and morphogenetic defects seen in MZ*stat3* gastrulae, as manifested by a shorter AP axis, as well as reduced AP dimensions of both axial and paraxial mesoderm cells, albeit larger in size, along the AP axis in these tissues (Fig 8). Moreover, cell intercalation seemed normal in MZ*stat3* embryos as evidenced by normal notochord width and the number of cells across the notochord at early segmentation (Fig 4B and 4C), as well as a single-cell column notochord formed subsequently in both WT and *stat3* mutant (S4 Fig). Therefore, we conclude that Stat3-mediated cell proliferation during blastula and gastrula stages promotes extension during zebrafish gastrulation, most likely by providing sufficient building blocks necessary for the ML intercalation-based AP extension (Fig 10A).

Consistent with this model, loss of *cdc25a* function in the zebrafish *standstill* mutant led to a bent and shorter body at 1 dpf [50]. We observed that ectopic *cdc25a* expression partially suppressed the extension phenotype in MZ*stat3* mutants and produced excess extension in WT gastrulae (Fig 9E and 9F). However, although trending, notochord AP dimension and number of notochord cells along AP axis were not significantly increased in WT embryos injected with *cdc25a* RNA. We attribute this to large variation between injections, suggesting that the axis elongation is sensitive to the dose of ectopic *cdc25a*. Whereas overexpression of Stat3 in MZ*stat3* could only rescue somite AP dimension and notochord cell number defects to M*stat3* level (the zygotic portion), reduction in adaxial cell number was fully normalized (Fig 7), suggesting a tissue-specific requirement of Stat3-dependent cell proliferation during morphogenesis.

The significance and novelty of the role cell proliferation plays during vertebrate gastrulation is further underscored by a recent publication providing evidence that cell division coupled with intercalations powers morphogenesis of chick epiblast before primitive streak formation [54]. Our studies in zebrafish demonstrate a key role of cell proliferation in producing sufficient number of cells needed for cell intercalations of mesenchymal cells that drive axial extension (Fig 10A).

### Stat3 is not required for planar cell polarity signaling during gastrulation

We gathered several lines of evidence arguing against Stat3 regulating C&E by promoting Wnt/PCP signaling and ML cell orientation [13]. First, the enlarged MZ*stat3* notochord cells, although rounder, exhibited normal ML orientation (Fig 4A and 4D–4H). Second, MZ*stat3* mutants displayed normal convergence of axial and paraxial tissues (Figs 4B, 4C, 3C and 3D). Third, we failed to detect any enhancement of cyclopia or axis extension phenotypes when zygotic *stat3* function was inactivated in Wnt/PCP pathway components mutants (Fig 4K and 4L), with a caveat that maternal *stat3* function was not removed in these experiments. In addition, cell division orientation of neuroectodermal cells shown to be regulated by Wnt/PCP signaling appeared normal in MZ*stat3* gastrulae (Fig 6E–6G).

However, our morphometric analyses implicate Stat3 in regulation of cell shape as MZ*stat3* notochord cells were rounder compared to WT with a bigger AP and a shorter ML dimension (Fig 4G and 4H, Fig 10B). One possibility is that the slightly reduced LWR is due to increased cell size. However, our observations support an alternative model where Stat3 plays a more direct role of Stat3 in cell shape regulation, as the enlarged cells resulting from the chemical inhibition of cell division increased in both AP and ML cell dimensions compared to WT cells (Fig 8J and 8K, Fig 10B). Indeed, in mouse keratinocytes and fibroblasts cytoplasmic Stat3 regulates microtubule and actin cytoskeleton through its interaction with Stathmin, a microtubule-destabilizing protein, and small Rho-GTPases, respectively [3, 55]. Given that inhibition of other regulators of Rho such as Rho kinase [56] and Gα12/13 heterotrimeric G proteins [57] impairs cell elongation during C&E, it will be interesting to investigate whether Stat3 utilizes similar mechanisms to shape gastrulating zebrafish cells.

### Stat3 loss-of-function as a tool to study scoliosis

We describe the first vertebrate *stat3* mutant being capable of surviving beyond embryonic stages, opening new avenues for functional studies of Stat3 in later developmental processes and disease. Before they perished as juveniles, *stat3* mutants exhibited scoliosis and excessive inflammation (Fig 2 and S2 Fig). Work from our and other laboratories linked early notochord malformations at embryonic and larval stages with scoliosis in juveniles and adults [58, 59]. However, *stat3* mutants showed normal notochord morphology during embryogenesis (Figs 4A, 3C and S4 Fig). Moreover, Alizarin Red staining at 15 dpf failed to reveal any structural abnormalities in *stat3* mutants in the notochord or differentiating vertebrae (between the swim bladder and the cloaca) (Fig 2D), indicating that the scoliosis phenotype in *stat3* mutant fish is likely not of congenital but of idiopathic type. As a key regulator of immune responses, abnormal Stat3 activity has been associated with immunodeficiency such as HIES in human [6] and Crohn’s disease-like conditions in mouse *Stat3* CKO [9]. With a global disruption of the *stat3* gene, our *stat3* mutant zebrafish warrants further characterization as a new candidate tool for studies of Stat3-related diseases in human.

In summary, we generated and characterized a valuable vertebrate *stat3* genetic model for further studies of development and disease. Our work provides direct evidence that cell proliferation promotes zebrafish axis extension, and clarifies the role of Stat3 in zebrafish C&E gastrulation movements as proliferation regulator, in part through Cdc25a activation. Further studies will verify whether cell cycle regulation function of Stat3 is conserved in larval and juvenile stages, and address the mechanisms underlying scoliosis and other phenotypes associated with *stat3* zebrafish mutations.

## Materials and methods

### Zebrafish strains and staging

Zebrafish are housed and handled under protocols approved by the Washington University Animal Studies Committee. AB* or AB*/Tubingen WT, *tri*^*vu67*^, *stat3*^*sa15744*^, and *slb*^*tz216*^ mutant zebrafish (*Danio rerio*) lines were used. Fish were normally fed with rotifers during larval stages followed by a mixture of rotifers and artemia during juvenile stages and adulthood. Some fish (as indicated in text) were fed exclusively with rotifers at all times to diminish food competition. Embryos were collected from natural matings, maintained in 28.5°C, and staged according to [18].

### Generation of *stat3* indel mutant lines

A TALEN pair was designed to target the boundary of Intron 4 and Exon 5 of the zebrafish *stat3* gene. The targeting sequences for the TALEN arms were 5’- TAACCTCTTACTCATCCTCCA -3’ and 5’-AAGAGGTTGTAGAAGTAGA-3’, respectively. An NlaIII restriction site within the 15-base pair long spacer between the two TALEN arms was used for assaying disruption of this sequence in genomic DNA (Fig 1A). TALEN constructs were assembled using the Golden Gate method [60] and used to generate indels in *stat3* target sequences as described [61]. Two alleles, *stl27* and *stl28*, containing a 7-base pair and a 2-base pair deletion in Exon 5, respectively, were originally confirmed by sequencing and identified using PCR-based genotyping (forward primer 5’-AGCTATTGCTTGGGTATAACCTCTTACTC-3’, reverse primer 5’-GCAGTCATACCTCCAGCACTC-3’, followed by NlaIII digestion). However, this genotyping method is not recommended to identify *stat3* mutation carriers as biased amplification of the mutant DNA possibly due to allelic competition during PCR may confound genotyping. Instead, we used allele-specific PCR amplification to identify *stl27* heterozygous and homozygous fish (shared forward primer 5’-CCACCTGTGACCATATGACTGAA-3’, WT allele reverse primer 5’-CTCCAACATCTTCATCTTCTGCTCCA-3’, *stl27* allele reverse primer 5’-CTCCAACATCTTCATCTTCTGTCCTG-3’). *stl27* and *stl28* alleles are predicted to encode truncated proteins of 158 and 168 amino acids, respectively.

### Plasmid construction, RNA synthesis and injection

Full-length coding sequence of zebrafish *stat3* was subcloned from the previously published *stat3* construct [35] into pCS2 plasmid and FLAG-tagged at the C-terminus. Full length coding sequence of zebrafish *cdc25a* was subcloned from *cdc25* Tol2 construct [30] into pCS2 plasmid. Capped RNAs were synthesized using mMessage mMachine kit (Ambion), and injected at 1- or 8-cell stage with doses specified in Results. 5 ng of *stat3* morpholino (MO1-*stat3*, http://zfin.org/action/marker/view/ZDB-MRPHLNO-051004-1) was injected at 1-cell stage as previously described [12].

### Whole-mount *in situ* hybridization and immunostaining

Embryos were fixed in 4% paraformaldehyde (PFA) in PBS. *In situ* hybridization was carried out as described [62]. Images were acquired and morphometric measurements were carried out manually with Fiji software.

Immunostaining was performed using a standard protocol. The following antibodies were used: anti-phospho-Histone H3 antibody (1:3,000, rabbit, Upstate, 06–570), and Alexa Fluor 488 or 568 goat anti-rabbit (1:500, Invitrogen). Embryos were counterstained with 4',6-diamidino-2-phenylindole (DAPI, 0.1 μg/mL, Invitrogen), mounted in 0.75% low melting temperature agarose (Lonza) in 0.3% Danieau’s solution (LMTD agarose), and imaged with the Quorum spinning disk confocal microscope (SDCM) using a 10x objective lens (10x). A Z-stack of over 200 μm was acquired at a step size of 3 μm and projected in Fiji. The number of nuclei was quantified using Analyze Particles plugin in Fiji.

### Western blotting

Five to six embryos (6 hpf) were deyolked and homogenized in a modified RIPA buffer [16]. Proteins were resolved in 4–12% NuPage Bis-Tris gels (Invitrogen) and transferred to PVDF membrane blocked with 10% milk in Phosphate Buffered Saline with Tween (PBS-T). Primary antibodies used were: anti-Stat3 (1:250, AnaSpec, 55861), anti-FLAG (1:1,000, Sigma-Aldrich, F1804), and anti-β-actin (1:1,000, Sigma-Aldrich, A5441). Secondary antibodies used were: donkey anti-mouse HRP (1:5,000, Fisher Scientific, SA1100) and goat anti-rabbit horseradish peroxidase (HRP) (1:5,000, Fisher Scientific, PR-W4011). Signals were detected with an enhanced chemiluminescence (ECL) kit (Perkin Elmer) and imaged using film.

### Quantitative Real-Time (qRT) PCR

Total RNA was isolated from 30–50 embryos with Trizol (Ambion) and treated with DNase (Zymo Research). For larvae and juveniles, the whole animals were subjected to snap freezing in liquid nitrogen and homogenized using a mortar and pestle. cDNA was synthesized using iScript kit (Bio-Rad). qRT-PCR was performed using CFX Connect Real-Time system and SYBR green (Bio-Rad), with at least three independent biological samples for each experiment. Primers are listed in S1 Table).

### Pharmacological treatment

WT embryos were dechorionated in 0.3x Danieau solution and incubated with 20 mM hydroxyurea (Sigma-Aldrich) and 150 μM aphidicolin (Sigma-Aldrich) in 4% dimethyl sulfoxide (DMSO, Sigma-Aldrich) from 5.7 hpf until desired stages [31]. Incubation in 4% DMSO was used as control.

### Morphometric analyses of live embryos

Embryos were injected with 200 pg *membraneEGFP* (*mEGFP*) RNA at 1-cell stage, mounted in 0.5% LMTD agarose at desired stages and imaged on Quorum SDCM with a 40x objective (N.A. 0.75). For cell body alignment and shape, image stacks were acquired and the top layer of the notochord cells were analyzed in Fiji [27]. To measure the AP dimension of the somite, five lines parallel to the notochord were drawn randomly in Fiji between two adjacent somitic furrows.

### Cell cycle imaging and analyses

For pre-MBT cell divisions, zygotes were injected within 20 minutes post-fertilization (mpf) with 70 pg *H2B-RFP* RNA and mounted in 0.3% LMTD agarose at 4–8 cell stage. Time-lapse movies were taken at 28.5°C with Quorum SDCM using a 10x objective lens. A z stack covering 200 μm at a 3–4 μm step distance was acquired every 1–2 min for at least 4 hours. Cell divisions were manually tracked in Fiji by quantifying the length from telophase to telophase. As H2B-RFP signal became clearly visible only from 8–16 cell stage, Cycles 5 (16 cells to 32 cells) to 9 (256 cells to 512 cells) were recorded and analyzed.

Post-MBT cell division experimental design was adapted with modifications from Dalle Nogare et al. [19]. To minimize inter-individual and experimental variability, we performed a combination of global and mosaic labeling, which allowed us to compare experiment and control lineages within the same embryo. At 1 cell stage, embryos were injected with *H2B-RFP* RNA. At 8-cell stage, one blastomere was injected with 18.8 pg (a dose equivalent to 150 pg at 1-cell stage) *membraneCherry* (*mCherry*) as control. An adjacent blastomere was injected with 18.8 pg *mEGFP* with or without 3.1 pg (a dose equivalent to 25 pg at 1-cell stage) *stat3-FLAG* (*stat3*-*F*) or *cdc25a* RNA. Time-lapse movies were recorded separately for mEGFP- (Fig 5B, see also S6 Movie) or mCherry- (Fig 5C, see also S7 Movie) clones of each embryo with Quorum SDCM and a 40x objective at 2-minute interval for the duration of 5–6 post-MBT cycles. At each time point, a z stack spanning 100 μm was acquired at a step size of 4 μm. Movies were converted to hyperstacks in Fiji. Cell divisions were manually tracked using MtrackJ plugin in Fiji.

### Cell division orientation analyses

The orientation of cell division of dorsal neuroectodermal cells was determined as previously described [63].

### Bone analyses

Juvenile fish were fixed in 4% PFA, bleached in 3% hydrogen peroxide/1% KOH, and stained with 1 mg/mL Alizarin Red in 1% KOH overnight for whole-mount bone staining. Soft tissues were cleared with 1% trypsin in 2% borax for up to a week. Larval vertebrae were stained *in vivo* by Alizarin Red (1 mg/mL) for 2 hours before imaging live on Quorum SDCM. Microcomputed tomography (Scanco uCT40) was used for 3D reconstruction and analyses of bone parameters (threshold set as ~150) of the juvenile vertebrae.

### Statistical analyses

WISH and immunostaining quantification, and morphometric analysis were performed blindly, followed by genotyping for the *stat3*^*stl27*^ allele. Data were collected in Excel (Microsoft), analyzed and graphed with GraphPad Prism (GraphPad Software). Student’s *t* test was applied to determine statistical significance (*p*<0.05) between two datasets. Kolmogorov-Smirnov test was used to compare angle distributions. All results are shown as Mean ± Standard Error of the Mean (SEM).

## Supporting information

S1 Table Nucleotide sequences of RT primers. * Four pairs of primers were used to detect *stat3* transcript. *stat3_RT* spans the deletion site in both *stl27* and *stl28* alleles; *stat3-RT1* amplifies a coding region upstream of the deletion site in all three splicing variants; *stat3_RT3* only amplifies a coding region downstream of the deletion site in the full length splicing variant; and *stat3-RT2* spans an alternative splicing site downstream of the deletion site, and detects two longer splicing variants. ** Two pairs of primers were used to detect *cdc25a* transcript in zebrafish embryos.
(DOCX)

S1 Fig Adult zebrafish *stat3* mutants develop late-onset scoliosis. (A-C) qRT-PCR showing *stat3* transcript levels using primers detecting various regions of *stat3* cDNA sequence in WT and MZ*stat3* embryos normalized to *gapdh*. (D) Western blot detecting total Stat3 in WT, MZ*stat3* and MZ*stat3* embryos overexpressing Stat3-F (50 pg RNA) at 6 hpf. *p<0.05, ***p<0.001, ****p<0.0001, error bars = SEM.
(TIF)

S2 Fig Various vertebral abnormalities contribute to the scoliosis phenotype in *stat3* animals. (A) Vertebrae of a WT larva at 29 dpf; anterior to the left. (B-H) Images of Alizarin stained skeletons showing various vertebral abnormalities. B and C, normal vertebral body and end plates, tilted intervertebral discs; D and E, bent vertebral body and non-perpendicular end plates; F-H, fractures and extra bony matrix. (I) Variations in larvae body length of *stat3* mutant and siblings at 22 dpf.
(TIF)

S3 Fig Injection of *stat3* morpholino results in dose-dependent axis extension defect. (A) *ntl* WISH in the notochord tissue in stage-matched control and *stat3* morphant embryos at 2-somite stage injected with various doses of MO1-*stat3* at one cell stage (lateral view, dorsal to the right, anterior to the top).
(TIF)

S4 Fig Convergence of the notochord into a single-cell wide column is not affected in M and MZ*stat3* embryos. (A-C) Confocal microscope image of 5-somite stage embryos in dorsal view, in which cell membranes are labeled with mGFP: WT (A), M*stat3* (B), and MZ*stat3* (C) (anterior to the top).
(TIF)

S5 Fig Pre-MBT cell divisions are lengthened in MZ*stat3* mutant embryos compared to WT. Depicted are confocal microscope snap-shots from a full pre-MBT cell cycle (Cycle 6, 32-cell stage to 64-cell stage) in WT and MZ*stat3* embryos from S phase to S phase.
(TIF)

S6 Fig Stat3 limits apoptosis during embryogenesis. (A-E) Apoptosis in WT (A, D) and MZ*stat3* (B, E) embryos at 10 hpf (A, B) and 11 hpf (D, E) detected by TUNEL labeling (dorsal view, anterior to the top). Number of TUNEL-positive cells are quantified in C and F. (G, H) *birc5a*/*survivin* (G) and *bcl2a* (H) transcript levels in WT and MZ*stat3* embryos at 1.5 hpf and 8.3 hpf determined by qRT-PCR. *p<0.05, **p<0.01, n.s. = non-significant, error bars = SEM.
(TIF)

S7 Fig Control experiments for post-MBT cell cycle analyses. (A) Experimental design for control post-MBT cell cycle analyses. Embryos labeled ubiquitously with H2B-RFP were mosaically injected with *memCherry* or *mGFP* mRNA at 8-cell stage for lineage tracing as described above. (B) Analyses of cell cycle lengths for Cycle 11–13 in WT embryos overexpressing mRNA or memCherry. Error bars = SEM.
(TIF)

S8 Fig Stat3 may regulate cell proliferation via transcriptional activation of other cell cycle regulators. (A) Maternal expression levels of cell cycle-regulating genes encoding zebrafish Cyclin A2, B1, B2, D1 and E in 16-cell stage WT and MZ*stat3* embryos detected by qRT-PCR. (B) *cdc25a* transcript level in mid-gastrula stage (8.3 hpf) MZ*stat3* and MZ*stat3* embryos injected with 25 pg of *stat3-F* RNA. **p<0.01, ****p<0.0001, n.s. = non-significant, error bars = SEM.
(TIF)

S9 Fig Effect of *cdc25a* overexpression in WT on notochord cell size and morphogenesis. (A and B) Dorsal view of 1-somite stage embryos showing cells labeled with mGFP: control embryos (A) and embryos injected with 25 pg to 50 pg *cdc25a* mRNA (B) (anterior to the top). (C-H) Analyses of notochord cells’ orientation (D), shape (E), long axis (length, F), short axis (width, G) and size (H) in A and B. (I and J) Confocal image of dorsal mesoderm in 3-somite stage control and *cdc25a*-overexpressing embryos labeled with mGFP with somite AP dimension illustrated with green arrow and somitic boundaries outlined in green (dorsal view, anterior to the top). (K-M) Quantification of somite AP dimension (K), numbers of adaxial cells (L) and notochord cells (M) in I and J. ****p<0.0001, error bars = SEM.
(TIF)

S10 Fig Onset of zygotic gene expression in MZstat3 and WT embryos. (A) Expression of *bozozok/dharma (boz)*, a zygotic target of *β-catenin*, in WT (top, animal and lateral view, AP, animal pole) and *MZstat3* embryos (bottom) at 4 hpf. Yellow arrows denote *boz* expression domain. (B) Expression of *floatinghead (flh)*, an early zygotic gene whose expression domain rapidly changes shape with convergence and extension of the axial mesoderm, in WT and *MZstat3* gastrulae in dorsal view and animal pole towards the top at 6 hpf. Yellow line indicates width of expression domain. (C) The mediolateral dimension of the *flh* expression domain at 6 hpf is not significantly different between WT and *MZstat3* embryos. n.s. = non-significant.
(TIF)

S1 Movie 3D rotation showing scoliotic spine in zygotic *stat3* larvae. Larvae were collected at stained with Alizarin Red. Z stacks were acquired on confocal microscope with a 10x objective. Images were processed with Fiji/ImageJ.
(AVI)

S2 Movie 3D rotation showing vertebrae with extra bony matrix in zygotic *stat3* larvae. Larvae were collected at stained with Alizarin Red. Z stacks were acquired on confocal microscope with a 10x objective. Images were processed with Fiji/ImageJ.
(AVI)

S3 Movie 3D rotation showing vertebral fracture in zygotic *stat3* larvae. Larvae were collected at stained with Alizarin Red. Z stacks were acquired on confocal microscope with a 10x objective. Images were processed with Fiji/ImageJ.
(AVI)

S4 Movie Confocal time-lapse movie of pre-MBT cell divisions (Cycles 5~9) in a WT embryo. Still images are displayed in S4 Fig. The embryo was ubiquitously labeled with H2B-RFP. Image of each time frame derives from z projection of a z stack covering 200 μm (animal view). Images were adjusted for level and contrast.
(AVI)

S5 Movie Confocal time-lapse movie of pre-MBT cell divisions (Cycles 5~9) in an MZ*stat3* embryo. Still images are displayed in S4 Fig. Images were acquired and processed as S1 Movie.
(AVI)

S6 Movie Confocal time-lapse movie showing post-MBT cell divisions of mEGFP-labeled lineage. Embryos were ubiquitously labeled with H2B-RFP and mosaically labeled with mEGFP. Illustrated in the movie is one z plane of the 100 μm confocal z stack captured at each time point.
(AVI)

S7 Movie Confocal time-lapse movie showing post-MBT cell divisions of mCherry-labeled lineage. Embryos were ubiquitously labeled with H2B-RFP and mosaically labeled with mCherry. Illustrated in the movie is one z plane of the 100 μm confocal z stack captured at each time point.
(AVI)
