# Supplementary material for: Stat3/Cdc25a-dependent cell proliferation promotes embryonic axis extension during zebrafish gastrulation
Source: PLoS Genet. 2017 Feb 21;13(2):e1006564. doi: 10.1371/journal.pgen.1006564 (PMC5319674; doi:10.1371/journal.pgen.1006564)
Supplement: S1 Table — * Four pairs of primers were used to detect stat3 transcript. stat3_RT spans the deletion site in both stl27 and stl28 alleles; stat3-RT1 amplifies a coding region upstream of the deletion site in all three splicing variants; stat3_RT3 only amplifies a coding region downstream of the deletion site in the full length splicing variant; and stat3-RT2 spans an alternative splicing site downstream of the deletion site, and detects two longer splicing variants. ** Two pairs of primers were used to detect cdc25a transcript in zebrafish embryos. (DOCX) [file pgen.1006564.s001.docx]

| Primer | Forward Sequence (5’-3’) | Reverse Sequence (5’-3’) |
| --- | --- | --- |
| *stat3_RT** | ACAGCAGGATGGCCAGGTTGC | TCTGTCTGGTGGCTGCTGCCT |
| *stat3_RT1** | CCCATGGAGCTCCGACAGTT | ACGATGCGGGCAATCTCCAT |
| *stat3_RT2** | TTTGGCAAATACTGCCGCCC | ATGAGAGAGTCGAGCGTGCG |
| *sta3_RT3** | CTGTGTCACCCCGTGTCCTT | GCCTCAGCAGCTTCGTTGTG |
| *il6* | ACGCGAATCTACAGCGTCCT | CACCTGCAGCTGGCTGTTTA |
| *tnfα* | ACCAGGCCTTTTCTTCAGGT | GCATGGCTCATAAGCACTTGTT |
| *bcl2a* | GAACTGGGGGCGGATCATTG | CCACGAAGGCATCCCAACC |
| *birc5a* | TGCACTCCAGAAAACATGGCT | ATCACAGCTGGGAGAATGCG |
| *cdc25a (pair1)*** | TCGCTCTCCTGCCTTCAAGA | GACAGCGAATGACAGGCGAA |
| *cdc25a (pair2)*** | TCCCTCCCGTTATGGAGTGT | GGGTGGTGGGGAGAGCATTA |
| *cdc25d* | AGCGAGCCATTAAGCGACTG | CATTGGATCCACCGCCTCTG |
| *ccna2* | GAGGCGCTAAACAGGGGTCT | AGGTGCTTTCTTGGAGCACG |
| *ccnb1* | GCAGCGAGAATCAGAACGCT | GGTGCAACCTTCACCTCCTTC |
| *ccnb2* | CATGGAAATGCACGCTCTGC | ACAACCTTCTTTGTCTGAACTGGT |
| *ccnd1* | TGGGATCTGGCCTCAGTGAC | TGAAGTTGACGTCTGTCGCAC |
| *ccne* | TCAGGGCTGAAGTGGTGTGA | GGAGTGAACCTTTCCCAGCC |
