# Supplementary figures and images for: Stat3/Cdc25a-dependent cell proliferation promotes embryonic axis extension during zebrafish gastrulation
Source: PLoS Genet. 2017 Feb 21;13(2):e1006564. doi: 10.1371/journal.pgen.1006564 (PMC5319674; doi:10.1371/journal.pgen.1006564)

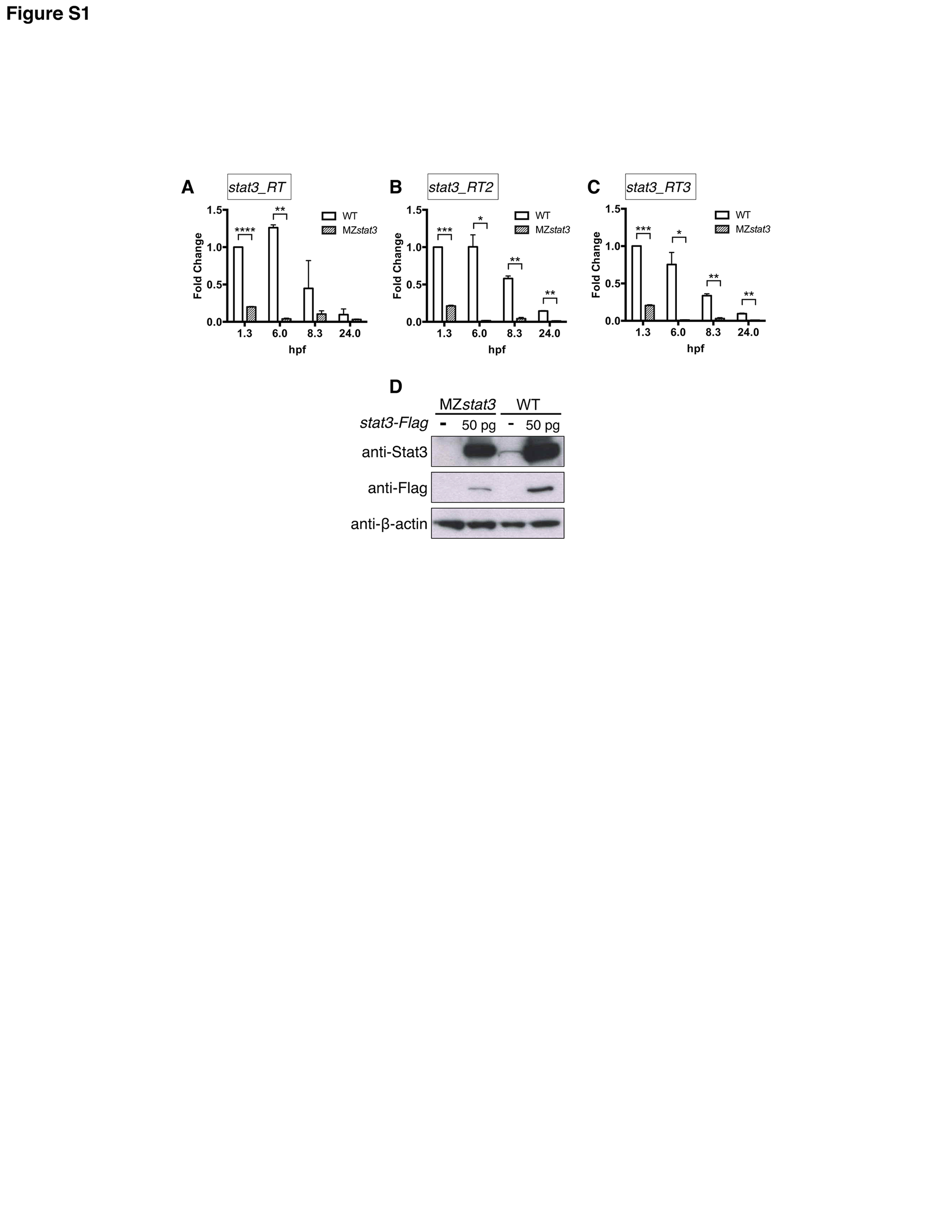

Supplement: S1 Fig — (A-C) qRT-PCR showing stat3 transcript levels using primers detecting various regions of stat3 cDNA sequence in WT and MZstat3 embryos normalized to gapdh. (D) Western blot detecting total Stat3 in WT, MZstat3 and MZstat3 embryos overexpressing Stat3-F (50 pg RNA) at 6 hpf. *p<0.05, ***p<0.001, ****p<0.0001, error bars = SEM. (TIF) [file pgen.1006564.s002.tif]

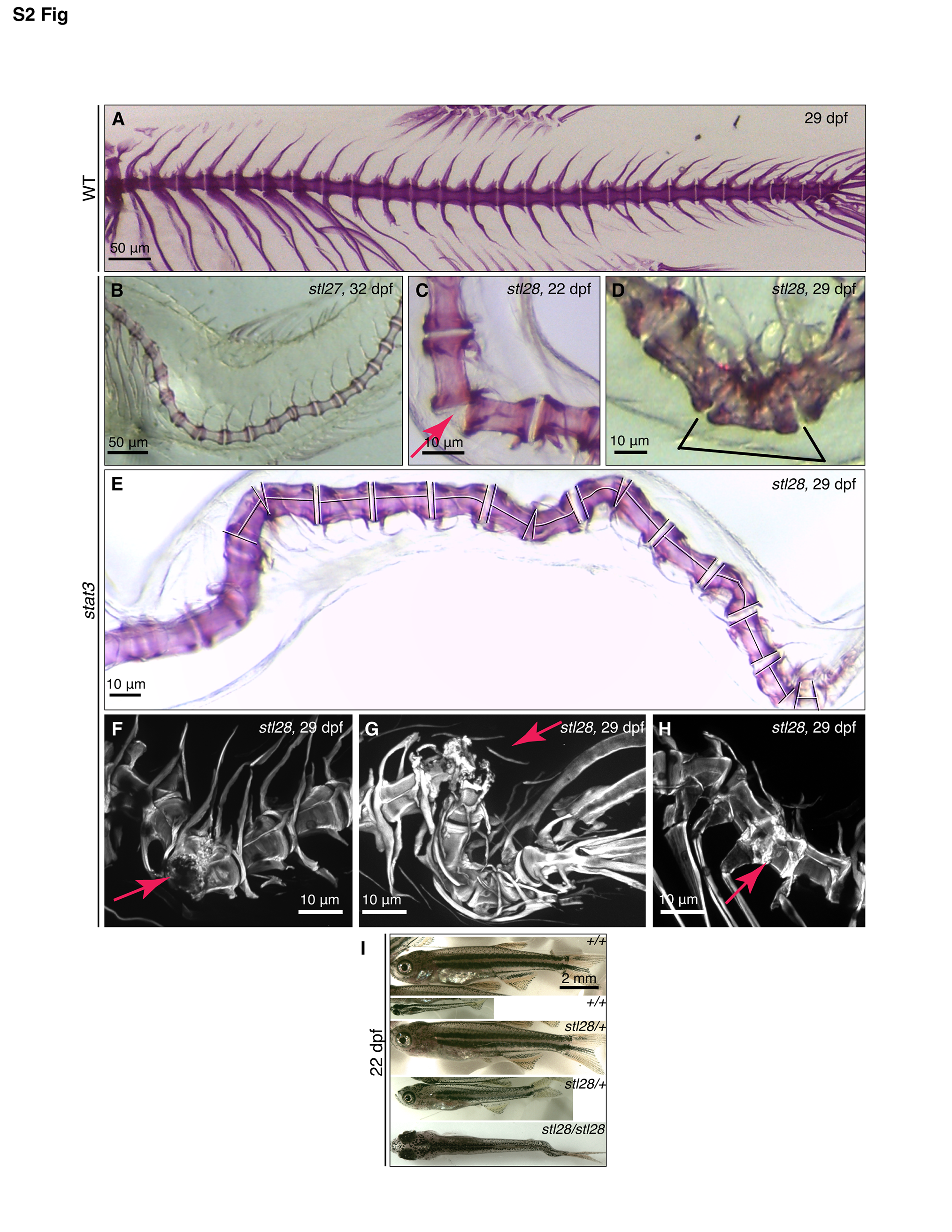

Supplement: S2 Fig — (A) Vertebrae of a WT larva at 29 dpf; anterior to the left. (B-H) Images of Alizarin stained skeletons showing various vertebral abnormalities. B and C, normal vertebral body and end plates, tilted intervertebral discs; D and E, bent vertebral body and non-perpendicular end plates; F-H, fractures and extra bony matrix. (I) Variations in larvae body length of stat3 mutant and siblings at 22 dpf. (TIF) [file pgen.1006564.s003.tif]

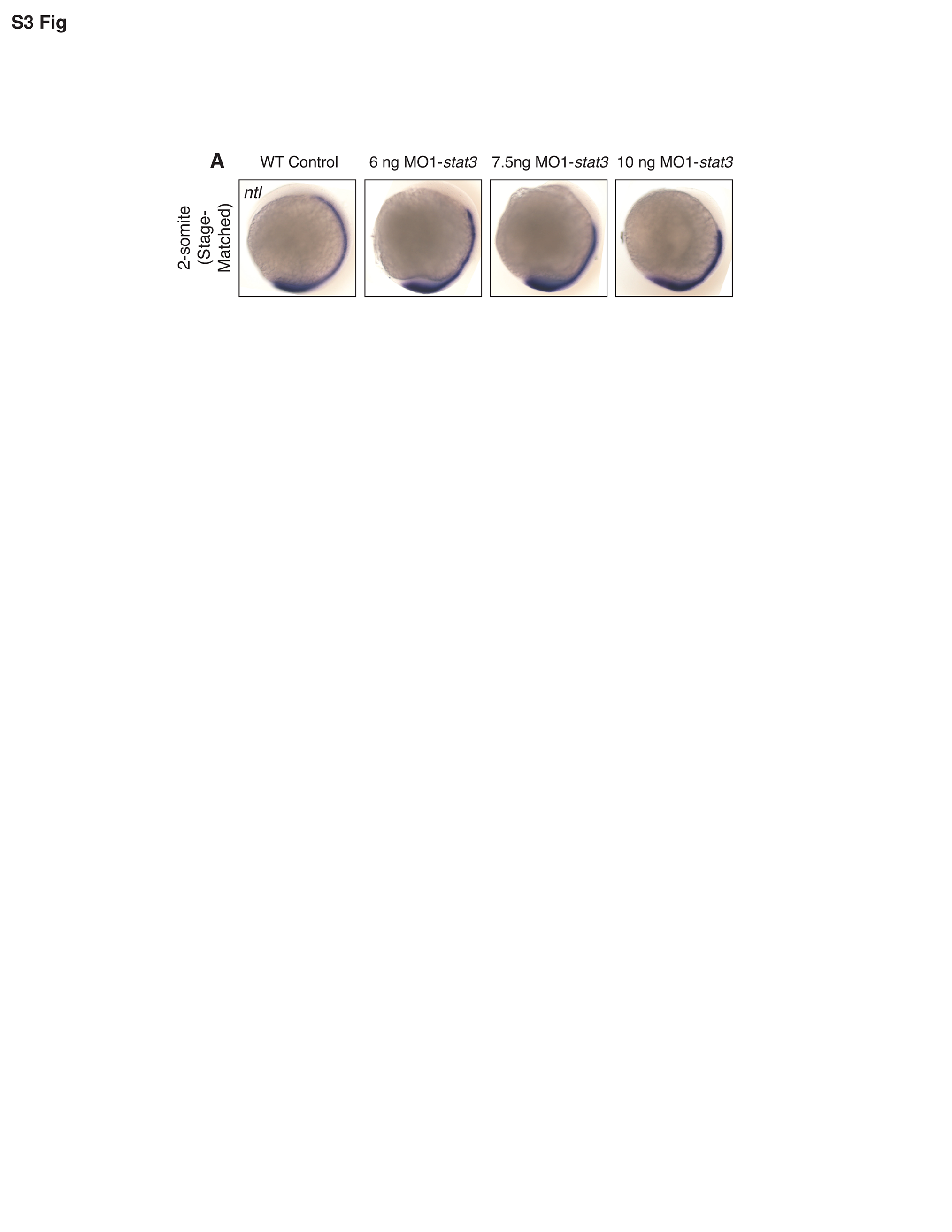

Supplement: S3 Fig — (A) ntl WISH in the notochord tissue in stage-matched control and stat3 morphant embryos at 2-somite stage injected with various doses of MO1-stat3 at one cell stage (lateral view, dorsal to the right, anterior to the top). (TIF) [file pgen.1006564.s004.tif]

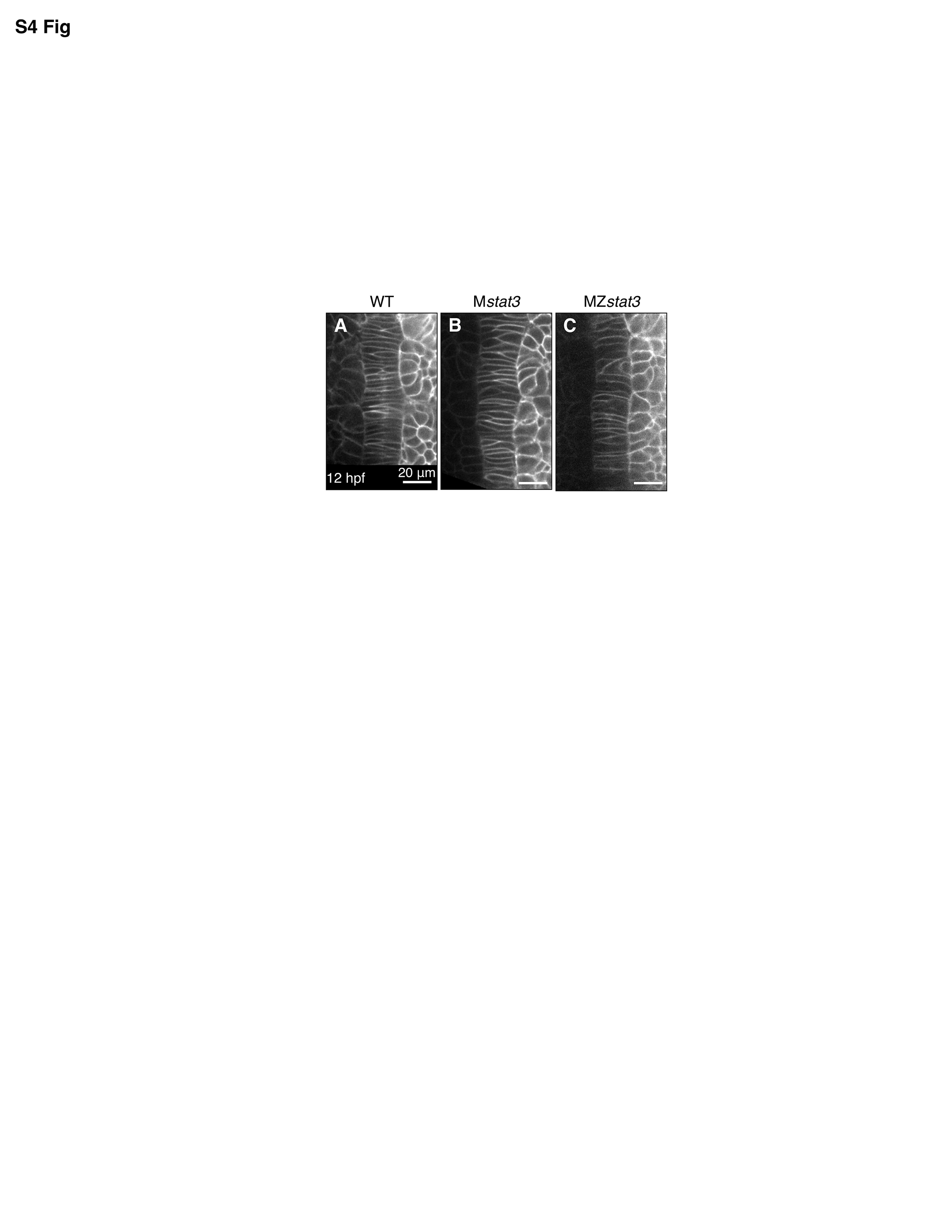

Supplement: S4 Fig — (A-C) Confocal microscope image of 5-somite stage embryos in dorsal view, in which cell membranes are labeled with mGFP: WT (A), Mstat3 (B), and MZstat3 (C) (anterior to the top). (TIF) [file pgen.1006564.s005.tif]

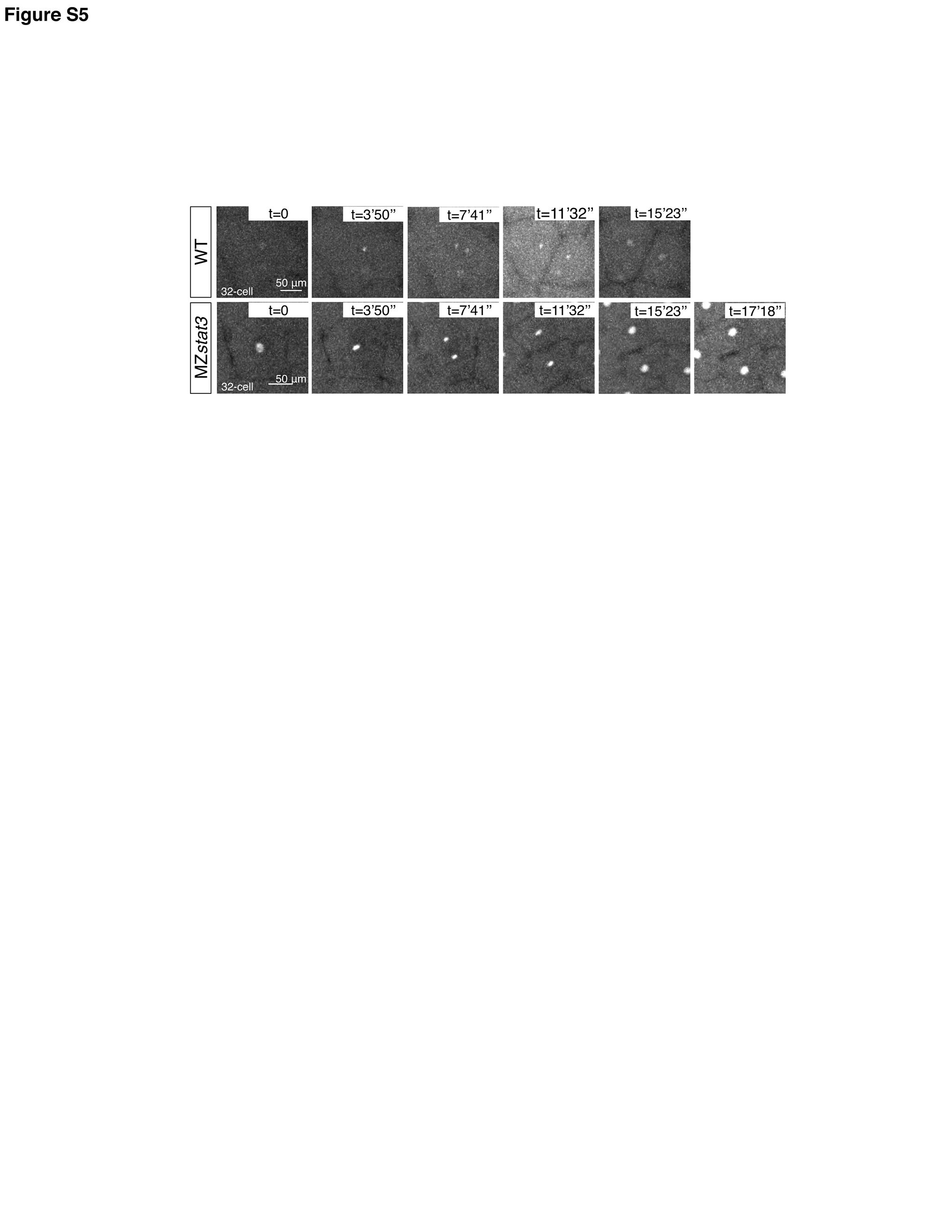

Supplement: S5 Fig — Depicted are confocal microscope snap-shots from a full pre-MBT cell cycle (Cycle 6, 32-cell stage to 64-cell stage) in WT and MZstat3 embryos from S phase to S phase. (TIF) [file pgen.1006564.s006.tif]

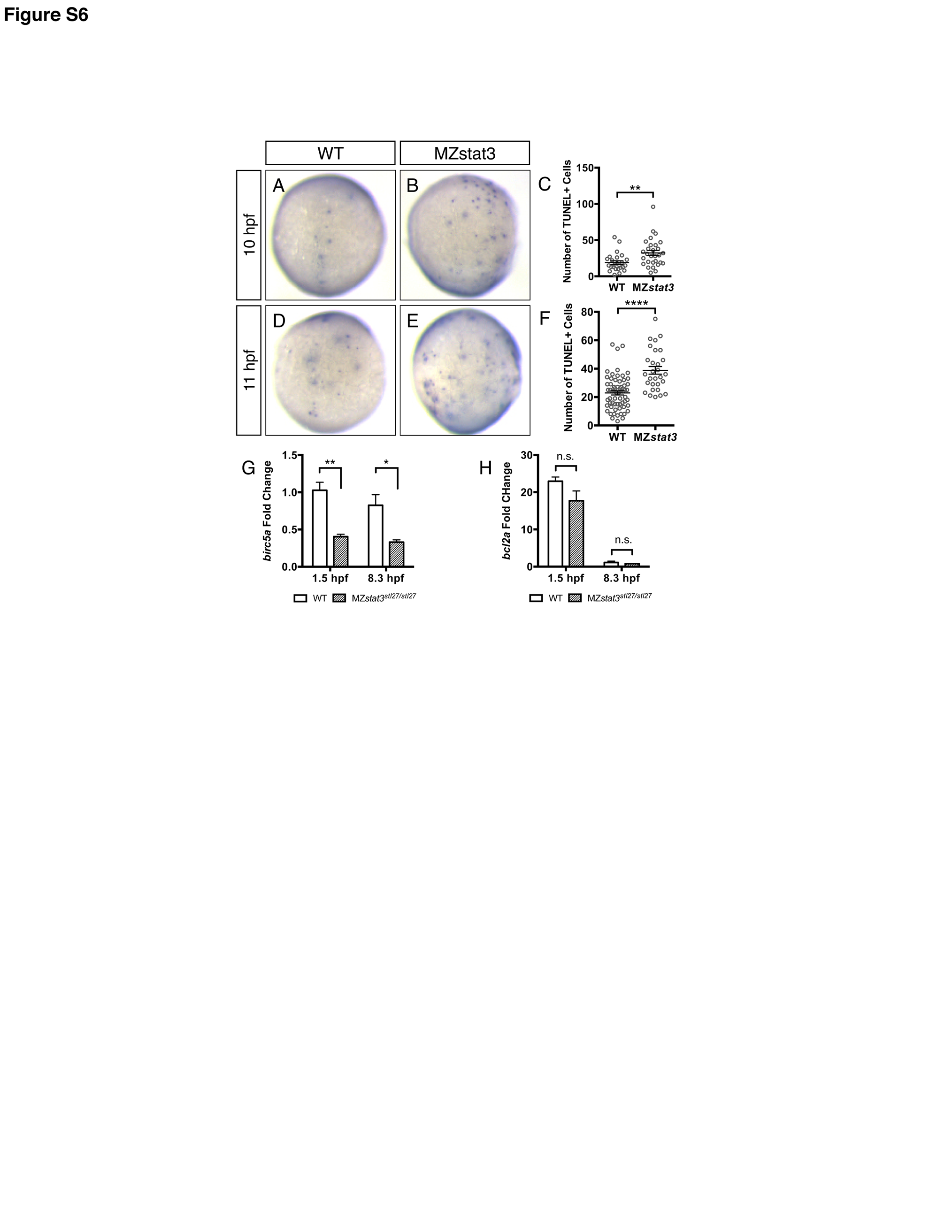

Supplement: S6 Fig — (A-E) Apoptosis in WT (A, D) and MZstat3 (B, E) embryos at 10 hpf (A, B) and 11 hpf (D, E) detected by TUNEL labeling (dorsal view, anterior to the top). Number of TUNEL-positive cells are quantified in C and F. (G, H) birc5a/survivin (G) and bcl2a (H) transcript levels in WT and MZstat3 embryos at 1.5 hpf and 8.3 hpf determined by qRT-PCR. *p<0.05, **p<0.01, n.s. = non-significant, error bars = SEM. (TIF) [file pgen.1006564.s007.tif]

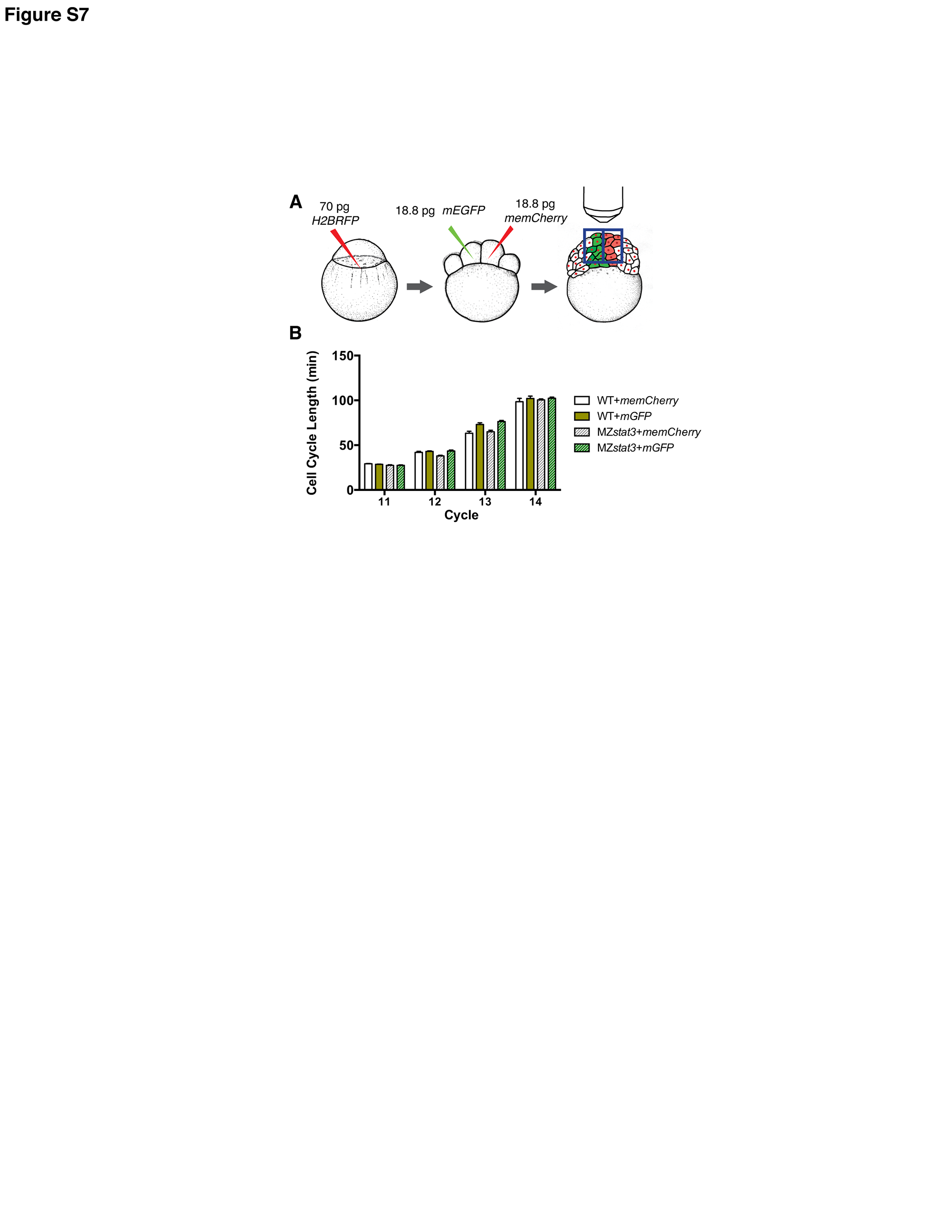

Supplement: S7 Fig — (A) Experimental design for control post-MBT cell cycle analyses. Embryos labeled ubiquitously with H2B-RFP were mosaically injected with memCherry or mGFP mRNA at 8-cell stage for lineage tracing as described above. (B) Analyses of cell cycle lengths for Cycle 11–13 in WT embryos overexpressing mRNA or memCherry. Error bars = SEM. (TIF) [file pgen.1006564.s008.tif]

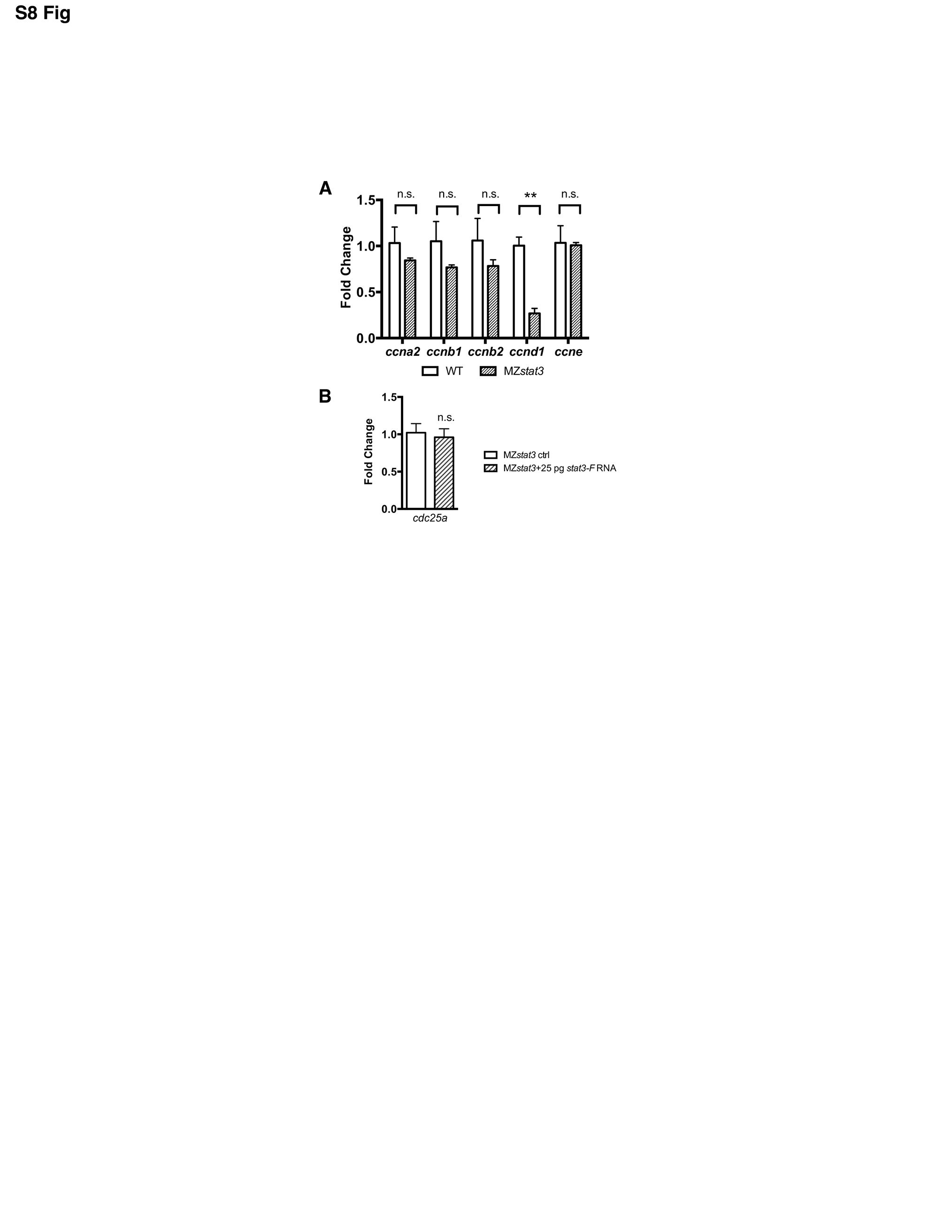

Supplement: S8 Fig — (A) Maternal expression levels of cell cycle-regulating genes encoding zebrafish Cyclin A2, B1, B2, D1 and E in 16-cell stage WT and MZstat3 embryos detected by qRT-PCR. (B) cdc25a transcript level in mid-gastrula stage (8.3 hpf) MZstat3 and MZstat3 embryos injected with 25 pg of stat3-F RNA. **p<0.01, ****p<0.0001, n.s. = non-significant, error bars = SEM. (TIF) [file pgen.1006564.s009.tif]

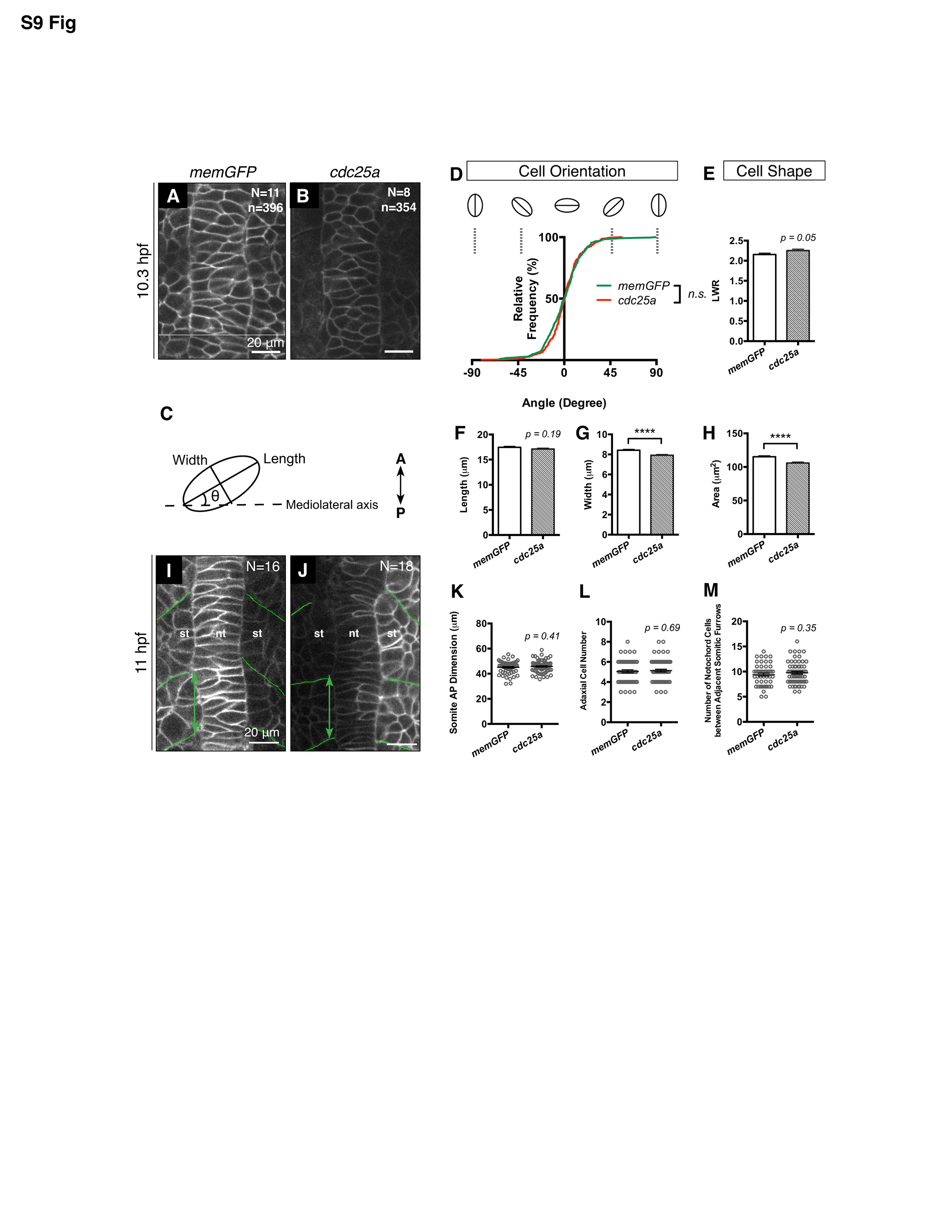

Supplement: S9 Fig — (A and B) Dorsal view of 1-somite stage embryos showing cells labeled with mGFP: control embryos (A) and embryos injected with 25 pg to 50 pg cdc25a mRNA (B) (anterior to the top). (C-H) Analyses of notochord cells’ orientation (D), shape (E), long axis (length, F), short axis (width, G) and size (H) in A and B. (I and J) Confocal image of dorsal mesoderm in 3-somite stage control and cdc25a-overexpressing embryos labeled with mGFP with somite AP dimension illustrated with green arrow and somitic boundaries outlined in green (dorsal view, anterior to the top). (K-M) Quantification of somite AP dimension (K), numbers of adaxial cells (L) and notochord cells (M) in I and J. ****p<0.0001, error bars = SEM. (TIF) [file pgen.1006564.s010.tif]

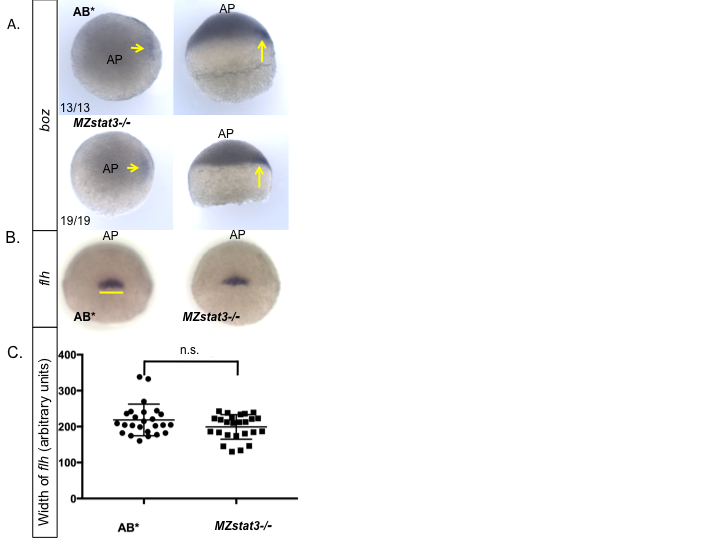

Supplement: S10 Fig — (A) Expression of bozozok/dharma (boz), a zygotic target of β-catenin, in WT (top, animal and lateral view, AP, animal pole) and MZstat3 embryos (bottom) at 4 hpf. Yellow arrows denote boz expression domain. (B) Expression of floatinghead (flh), an early zygotic gene whose expression domain rapidly changes shape with convergence and extension of the axial mesoderm, in WT and MZstat3 gastrulae in dorsal view and animal pole towards the top at 6 hpf. Yellow line indicates width of expression domain. (C) The mediolateral dimension of the flh expression domain at 6 hpf is not significantly different between WT and MZstat3 embryos. n.s. = non-significant. (TIF) [file pgen.1006564.s011.tif]
